# Circulating neuron-derived cfDNA for blood-based detection of Alzheimer’s and other neurodegenerative conditions

**DOI:** 10.3389/fneur.2026.1822479

**Published:** 2026-08-06

**Authors:** Chad Pollard, Ryan Miller, Isaac Stirland, Mykle Keni, Andrew Jenkins, Erin Saito, Jonathon T. Hill, Tim Jenkins

**Affiliations:** 1 Department of Cell Biology & Physiology, Brigham Young University, Provo, UT, United States; 2 Resonant, LLC, Pleasant Grove, UT, United States; 3 Department of Biology, Brigham Young University, Provo, UT, United States

**Keywords:** Alzheimer’s disease, blood biomarkers, cell-free DNA, liquid biopsy, methylation, mild cognitive impairment, neurodegeneration, precision medicine

## Abstract

Blood-based biomarkers for neurodegenerative diseases are improving early detection and staging, but current assays primarily reflect aggregate neuropathology or generalized neuronal injury and do not resolve the specific neuronal populations affected. Circulating cell-free DNA (cfDNA) retains stable DNA methylation patterns reflective of tissue and cellular origin, making it a promising substrate for cell-of-origin analysis. However, conventional methylation approaches are limited by bisulfite-associated DNA damage and amplification-related bias, hindering the detection of neuron-derived cfDNA, a small fraction of total circulating cfDNA. Here, we present proof-of-concept evidence that native nanopore sequencing can support both brain methylation atlas generation and downstream cfDNA cell-of-origin classifier development in neurodegenerative disease. By directly profiling endogenous DNA methylation without bisulfite conversion or PCR amplification, nanopore sequencing preserves native molecules, reduces processing-related bias, and enables flexible, genome-wide methylation profiling that can be iteratively expanded as additional reference cell types are incorporated. Using whole-genome native nanopore sequencing, we generated a methylation reference atlas from six primary human neural cell populations—cortical neurons, dopaminergic neurons, spinal motor neurons, astrocytes, Schwann cells, and microglia—and developed cell-type-informed cfDNA classifiers. Classifier performance was assessed in silico using dilution series designed to model physiologic admixture. The framework was then applied to 137 blood plasma samples from individuals with mild cognitive impairment (MCI), Alzheimer’s disease (AD), Parkinson’s disease (PD), amyotrophic lateral sclerosis (ALS), and healthy controls. Elevated circulating cfDNA fragments exhibited methylation patterns similar to reference profiles from selectively vulnerable neuronal populations, including cortical neuron-like signatures in AD and progressive MCI, dopaminergic neuron-like signatures in PD, and spinal motor neuron-like signatures in ALS. Multivariate integration of neuronal signatures improved the separation of diagnostic groups within this cohort (AUC > 0.85). Although the reported atlas is limited and additional validation in larger and independent cohorts will be required, these results support the feasibility of native cfDNA nanopore methylation sequencing as a flexible platform for brain-derived cfDNA analysis and more cell-type-informed investigation of neurodegeneration from peripheral blood.

## 1 Introduction

Circulating cell-free DNA (cfDNA) has emerged as a complementary biomarker class with the potential to address important gaps in the current blood-based biomarker landscape for neurodegenerative diseases (1–5). Existing blood-based assays detect markers of disease-associated neuropathology, including amyloid burden, tau pathology, as well as generalized neuronal injury, such as NfL and GFAP (6–10). However, these markers provide limited information about which brain regions are affected, how selective vulnerability evolves over time, or how rapidly neuronal loss is occurring across distinct neuroanatomical systems. Greater cellular and anatomic resolution could strengthen neurodegeneration research, with potential to support earlier diagnosis, improved clinical trial stratification, and more biologically informed (2) monitoring of disease progression and therapeutic response (11).

DNA methylation provides a strong biological foundation for this effort. As a stable regulator of gene expression and a defining feature of tissue and cell identity, methylation is maintained in cell-type-specific patterns that persist in cfDNA fragments released into circulation (12–15). These properties have made methylation a particularly useful substrate for tissue- and cell-of-origin analysis, especially as differentially methylated regions (DMRs) capable of distinguishing cell lineages have become increasingly defined (16–18). More broadly, reference-based deconvolution of mixed tissues and bulk biological matrices is now well established, with most cell-of-origin studies largely relying on methylation arrays, bisulfite sequencing, and related methylation profiling approaches (19).

Although these strategies have expanded across oncology, organ transplantation, and prenatal testing to detect cfDNA released from injured or dying cells into circulation (17, 18, 20, 21), neurodegenerative disease remains a comparatively underexplored domain for cfDNA analysis (22). Recent studies have reported increases in total cfDNA in acute brain injury and related neurological conditions in blood and cerebrospinal fluid (CSF), supporting that brain cell death can be monitored through circulating nucleic acids (4, 23, 24). Neuronal- and glial-associated cfDNA signals have also been shown to distinguish neurological and psychiatric conditions and to retain information related to brain region or cell-type origin (1, 2, 4, 25, 26).

Together, these studies support the broader feasibility of cfDNA methylation for cell-of-origin analysis in neurological disease, but they also highlight several persistent technical challenges. In neurodegenerative disease, neuron-derived cfDNA is expected to represent a very small fraction of total circulating cfDNA, estimated at approximately 2% (17). Tissue-of-origin inference in this context is therefore constrained by ultra-low DNA input, sparse genomic coverage, and a non-neuronal background derived primarily from blood and other peripheral tissues. In addition, many foundational methylation workflows rely on bisulfite conversion and amplification, which can introduce DNA damage, obscure other epigenetic marks, and introduce biases, especially with limited cfDNA inputs (27–29). These constraints are particularly relevant when attempting to resolve subtle, low-abundance neuronal signals from plasma or other body fluids.

Our prior pilot work identified elevated cortical neuron-derived cfDNA in individuals with Alzheimer’s disease (AD) and mild cognitive impairment (MCI) relative to healthy controls using a targeted methylation assay (30). While that study provided initial evidence consistent with other cfDNA studies of the brain (31), it was similarly limited by locus restriction and the technical constraints of targeted bisulfite-based profiling.

Native nanopore sequencing offers a potential solution to these limitations by directly profiling endogenous DNA methylation without chemical conversion or amplification, thereby preserving native cfDNA molecules and reducing associated biases (27, 28, 32). Emerging work further demonstrates the feasibility of low-coverage nanopore cfDNA profiling in liquid biopsy settings (33–36). Beyond methylation, nanopore sequencing can simultaneously capture additional features from native cfDNA, including fragmentomic patterns and structural variants such as copy number alterations, which have been shown to be informative for detecting cell-type- and cancer-associated signals (34).

Building on these capabilities and prior methylation-based work, we tested whether native cfDNA nanopore methylation sequencing can recover cell-type-associated signals aligned with selectively vulnerable neuronal populations in peripheral blood beyond cortical neurons in AD. The goal of the present study was to provide proof of concept that native nanopore sequencing can support both reference atlas generation and downstream classifier development for cfDNA methylation analysis in neurodegenerative disease. Although other brain cell reference atlases have also been built from cfDNA fragmentomics (37) and RNA-seq-based transcriptomic profiles (38, 39), methylation remains one of the most stable and widely used substrates for cell-of-origin deconvolution (19). Here, we specifically extend that framework by generating a primary human brain methylation atlas of several neuronal and glial cell types using native whole-genome nanopore sequencing and applying derived classifiers to plasma samples from individuals with AD, MCI, Parkinson’s disease (PD), and amyotrophic lateral sclerosis (ALS).

## 2 Materials and methods

### 2.1 Sample acquisition

#### 2.1.1 Purified neural cell types

Purified human neurons and glial cells were obtained from commercial vendors (Accegen and Creative BioArray; Supplementary Table 1). Cell types included both primary and induced pluripotent stem cell (iPSC)-derived cortical and dopaminergic neurons, as well as primary astrocytes, Schwann cells, and microglia.

Postmortem human spinal cord tissue was procured through BioIVT under institutional protocols for deidentified human biospecimen research (BioIVT ASTERAND® Normal Human Tissue). Upon receipt, the tissue was rapidly processed to preserve DNA integrity. Spinal motor neurons were isolated using a Percoll gradient method adapted from previous methods (40), and optimized for mature human spinal cord tissue. Briefly, tissue was mechanically dissociated and layered onto dual isosmotic Percoll gradients (35 and 12.5%). Samples were centrifuged at 800 g for 30 min, after which the 12.5% layer was collected for downstream processing. This fraction was enriched for spinal motor neurons and used for subsequent DNA extraction and methylation profiling. Optimization steps included adjusting gradient concentrations, centrifugation parameters, and handling conditions to account for the increased density and fragility of mature human neurons.

Genomic DNA extracted from these purified populations were used to generate whole genome nanopore methylation reference profiles for classifier training, evaluate cell type specificity, and validate the performance of methylation-based models for cfDNA deconvolution.

#### 2.1.2 Human plasma samples

A total of 219 human plasma samples (1 mL each) were obtained from BioIVT (Supplementary Table 2, Westbury, NY, USA), representing six clinically confirmed diagnostic categories: Alzheimer’s disease (AD; *n* = 45), mild cognitive impairment (MCI; *n* = 5), Parkinson’s disease (PD; *n* = 64), sporadic amyotrophic lateral sclerosis (SALS; *n* = 65), familial amyotrophic lateral sclerosis (FALS; *n* = 10), and cognitively normal healthy controls (HC; *n* = 30). All samples were accompanied by case report forms (CRFs), informed consent documentation, and associated clinical metadata. Demographic variables included sex, ethnicity, and age, defined as age at the time of blood collection. Available clinical measures included Mini-Mental State Examination (MMSE) scores at multiple timepoints and cerebrospinal fluid (CSF) concentrations of disease-associated protein biomarkers. These included Aβ1-42, Aβ1-40, Aβ1-38, total tau, *α*-synuclein, and PARK7, quantified using validated commercial assays (Meso Scale Discovery [MSD], INNOTEST, and Covance platforms).

To reduce potential confounding from demographic variables known or suspected to influence methylation (17, 22, 41), plasma samples were selected to be as closely matched as possible across diagnostic groups for age, sex, and ethnicity. All BioIVT specimens were de-identified prior to receipt and used in accordance with institutional review protocols and ethical guidelines governing secondary use of human biological materials. Collection site information was not recoverable for the disease samples obtained from BioIVT. However, healthy control plasma samples were known to have been collected across five distinct sites. To minimize potential technical bias during downstream sequencing, samples were randomly distributed across flow cells, with 24 samples per flow cell, so that disease and control groups were not processed in single, isolated batches. Potential technical structure related to flow cell, age, and sex was assessed by PCA of plasma methylation features (Supplementary Figure 1).

Clinical plasma samples were progressively filtered for downstream analysis. Samples were first required to report diagnosis, processing batch ID, and at least 25 in-region reads, yielding 183 samples. Reported sex was then cross-checked using X chromosome copy number analysis. Samples with discordant assignments, defined as fewer than 1.5 X chromosomes in reported females or more than 1.5 X chromosomes in reported males, were excluded, leaving 179 samples. To avoid pseudoreplication, replicate samples from the same donor were collapsed by retaining the single sample with the highest total read count, removing 41 replicates and yielding 138 samples. Samples with missing age were excluded, and healthy controls younger than 30 years of age were removed from further analyses. After final filtering, 137 samples were retained for downstream clinical analyses (Supplementary Table 2). Demographic characteristics of the final analytic cohort, including control-versus-case comparisons, are reported in Supplementary Table 3. Values are summarized as mean ± SD for age and n (%) for categorical variables, with *p*-values calculated using Welch’s two-sample t-test for age and Fisher’s exact test for sex and ethnicity.

### 2.2 DNA processing

All biospecimens were processed by Wasatch BioLabs (WBL), a high-throughput sequencing provider specializing in clinical and research applications of Oxford Nanopore Technologies sequencing. Sample processing followed standardized internal quality control protocols and was managed through a laboratory information management system (LIMS) to ensure traceability and data integrity.

#### 2.2.1 Genomic DNA

Genomic DNA (gDNA) was extracted from purified cell types using the DNeasy Blood & Tissue Kit (Cat # 69504) and libraries were prepared according to Wasatch Biolabs’ (WBL’s) Direct Whole Methylome Sequencing (dWMS) protocol, a modified whole genome ONT sequencing service optimized for methylation analysis. Extracted DNA was fluorometrically quantified using the Qubit dsDNA High Sensitivity Assay (Thermo Fisher Scientific) and assessed for purity and integrity using the NanoDrop spectrophotometer (Thermo Fisher Scientific) and Agilent TapeStation 4,200 (Agilent Technologies), respectively.

Previous studies have shown that with nanopore long-read sequencing, genomic coverage as low as 10x is sufficient to accurately recapitulate 5mC methylation frequencies obtained by methylation microarray, digital restriction enzyme analysis, and reduced representation bisulfite sequencing (RRBS) (42). Following gDNA dWMS sequencing, cell types included in model training achieved an average of 15x coverage, ensuring sufficient coverage for methylation signal detection and accurate cell-type classification.

#### 2.2.2 Cell-free DNA

cfDNA was extracted from human plasma samples using either the MAGicBead cfDNA Isolation Kit (Zymo, Cat # D4086) or the QIAmp Circulating Nucleic Acid Kit - cfDNA/cfRNA Isolation (Qiagen, Cat # 55114). cfDNA library preparation was performed according to WBL’s Direct Methylation Sequencing (dMS) for cfDNA service, an nanopore-based cfDNA sequencing service for unbiased, native cfDNA sequencing. cfDNA quantity was measured using Qubit fluorometric quantitation (Thermo Fisher Scientific), and sample purity and integrity were assessed using NanoDrop spectrophotometry and the Agilent TapeStation 4,200, respectively, as described above for gDNA. While a minimum of 10 ng of cfDNA was required for downstream library preparation, cfDNA extracted from human blood plasma samples (1 mL) yielded an average 21.2 ng total cfDNA, consistent with reported ranges and disease-associated elevations in circulating cfDNA (43, 44).

### 2.3 DNA sequencing

DNA methylation array data for purified cortical neurons, dopaminergic neurons, and iPSC-derived spinal motor neurons were generated at the University of Utah using the Illumina Infinium MethylationEPIC v2 array platform in alignment with manufacturer protocols.

gDNA and cfDNA libraries were sequenced on the ONT PromethION P24 platform (Oxford Nanopore Technologies) using MinKNOW v24.02.19. Raw signal data (POD5 files) were basecalled with the dna_r10.4.1_e8.2_400bps_hac@v4.3.0_5khz model with 5mCG_5hmCG modified base calling activated. Resulting reads were processed with samtools (merge, sort, and index)^1^ and aligned to the GRCh38 human reference genome using Dorado.^2^ Modified base calls were then extracted using ONT’s modkit software^3^ to generate per-read methylation summary files.

### 2.4 Differential methylation analysis and classifier development

All array samples underwent standard preprocessing and normalization. Raw IDAT files were processed using the minfi R package (45). Beta values representing the fraction of methylation at each CpG site were obtained following SWAN (Subset-quantile Within Array Normalization). To assess data quality, beta value density plots were examined to confirm the expected bimodal distribution, characterized by peaks in the unmethylated (0.0–0.2) and methylated (0.8–1.0) ranges, with a flat valley in the intermediate methylation range. Samples failing to meet these distribution criteria were excluded, and the remaining samples were re-normalized using the same procedures.

For all nanopore sequencing data. CH3 methylation files were created for each sample and processed with ModSeqR (46), an open-source R package for. CH3 database generation, region/window summarization, differential methylation testing, and quality control visualization (v0.8.0).^4^ Methylation databases were indexed by individual CpG position using the make_pos_db() function. Using the summarize_windows() function, methylation data were summarized into 200-bp windows containing at least 5 CpGs. This threshold was selected to accommodate cfDNA fragment length while retaining as many informative regions as possible for classifier development, since requiring more CpGs would substantially reduce the number of usable regions, particularly under shallow sequencing conditions. Differential methylation analyses were then performed between each neural cell type and pooled human blood plasma using the calc_mod_diff() function. Differentially methylated regions (DMRs) were identified by collapsing adjacent windows with the collapse_windows() function and retaining regions with a minimum methylation delta of 0.8 between the target cell type and blood plasma.

To generate cell-type-specific classifiers, DMRs identified for each target population were further filtered to remove regions that overlapped with DMRs from other neural reference populations. This yielded a non-overlapping set of classifier DMRs for each cell type. Classifier development was performed independently of the clinical disease cohort. DMR discovery and classifier construction used only the reference cell types and the pooled plasma background. Thus, classifier inputs were restricted to cfDNA reads overlapping these cell-type-specific DMRs, and no broader feature set outside the DMR framework was used. Pooled plasma was procured from BioIVT and used as the comparison background reference. Although the exact number of contributing individuals was not provided, the vendor indicated that each pooled sample was derived from hundreds of healthy donors spanning a range of ages, sexes, and ethnic backgrounds.

### 2.5 Read-level classification and cfDNA fraction estimation

Read-level classification was performed using the classify_reads() function in ModSeqR (46). For each read overlapping a classifier DMR, methylation concordance was calculated relative to the reference methylation pattern of the target cell type and the pooled plasma background. Reads were assigned to the target cell type or to blood only if they met a predefined concordance threshold of 99%. Reads failing to meet this threshold for either class were labeled “unknown” and excluded from downstream fraction calculations. Reported cfDNA fractions, therefore, represent fractions of informative, classifiable DMR-overlapping reads rather than fractions of all sequenced cfDNA molecules.

For each classifier, the reported cell-type fraction was calculated as the number of reads assigned to the target cell type divided by the total number of reads assigned either to that cell type or to blood across the corresponding DMR set. For example, the cortical neuron-derived cfDNA fraction was calculated as:

$$\text{Cortical Neuron Fraction}=\frac{\text{Cortical Assigned Reads}}{\begin{array}{l} \text{Cortical Assigned Reads}+\\ \text{Blood assigned Reads} \end{array}}$$

Equivalent calculations were performed for dopaminergic neurons, spinal motor neurons, and other cell-type classifiers. Reads not overlapping classifier DMRs, as well as DMR-overlapping reads classified as unknown, were omitted from these calculations.

### 2.6 *In Silico* analytical validation

To evaluate analytical performance, we generated in silico mixtures of cell-type-specific DNA in a background of plasma-derived cfDNA (Supplementary Table 4). Genomic DNA from each purified reference cell type was computationally fragmented to approximate cfDNA fragment size distributions (Supplementary Figure 2), producing cell-derived read profiles more comparable to patient plasma cfDNA. Using these fragmented reads, we constructed synthetic datasets containing approximately 1,000,000 total reads with varying proportions of cell-specific reads spiked into healthy donor plasma cfDNA. The dilution series included nine fractional compositions: 0, 0.1, 1, 10, 15, 25, 50, 75, and 100% target cell-type reads, with the remaining balance composed of plasma-derived reads (Supplementary Table 4). We did not systematically evaluate classifier performance below 0.1%; this remains an important area for future study given the low expected abundance of neuron-derived cfDNA in plasma.

At each dilution point, the relevant DMR-based classifier was applied to the synthetic mixtures using the same read-level classification framework described above. Predicted fractions were generated by aggregating read-level assignments and were compared with the known spiked-in proportions. Classifier performance was assessed by linear regression and the coefficient of determination (R^2^), providing measures of concordance and linearity across the dilution range (Supplementary Figures 3–11). Specificity was further evaluated by applying each classifier to DNA from unrelated reference cell types and to iPSC-derived neuronal alternatives. We did not systematically vary the number of DMRs or CpGs used per classifier in the present study; optimization of classifier performance as a function of DMR set size is an important future direction.

### 2.7 Clinical statistical analysis

Clinical analyses were performed as an initial proof-of-concept evaluation of whether classifier-derived cfDNA signals could identify neuron-derived cfDNA in patient samples and distinguish diagnostic groups. For each cell type of interest, cfDNA classification fractions were first visualized across diagnostic categories using boxplots with overlaid individual data points.

To compare cell classification signals between diagnostic groups in the clinical plasma cohort, the Wilcoxon rank-sum test (Mann–Whitney U test), a rank-based nonparametric test originally developed for pairwise comparisons and widely used as an alternative to the two-sample t-test when distributional assumptions are not well satisfied (47), was used. In methylation studies, this test is well-suited to continuous measures such as methylation-derived classification scores because it compares group distributions without requiring normality, making it robust to the distributions commonly observed in methylation data, including bounded *β*-value behavior and heterogeneity in variance (48). We therefore applied two-sided Wilcoxon rank-sum tests to compare cell-type-associated cfDNA classification scores between independent clinical groups. To account for the multiple pairwise comparisons performed across classifiers and diagnoses, raw *p*-values were adjusted using the Benjamini-Hochberg procedure, which controls the false discovery rate and is a standard approach for high-dimensional methylation analyses (49). Adjusted *p*-values < 0.05 were considered statistically significant. All analyses were performed in R (v4.4.1) using wilcox.test() from base R and p.adjust().

For predictive modeling, logistic regression classifiers were built for each disease-versus-control comparison. To assess whether cfDNA methylation features contributed discriminatory signal beyond demographic and technical variables alone, three model classes were evaluated in parallel: methylation-only, covariates-only, and combined models (Supplementary Figure 12). Covariates included age, sex, and flow cell. For the original split-sample analysis, the dataset was partitioned into training (30%) and test (70%) sets using stratified sampling to preserve class balance. Models were trained using one or more cfDNA classifier fractions as predictors. For single-classifier analyses, a single predictor was used; for multivariate analyses, multiple classifier fractions were combined. The optimal decision threshold was identified in the training set using Youden’s J statistic (50) from the ROC curve and then fixed for evaluation in the held-out test set.

To assess the stability of classifier performance beyond a single train-test split, we used the train() function in the caret package to perform repeated 10-fold cross-validation for each disease-versus-control logistic regression model. This was applied identically to the methylation-only, covariates-only, and combined models to enable equivalent comparisons and improve model stability (Supplementary Figure 12). For each model, the data were split into 10 folds and the procedure was repeated 20 times. In each iteration, a logistic regression model was trained on 90% of the data and used to generate predictions on the held-out 10%. ROC curves and associated performance metrics were computed exclusively from these held-out, out-of-fold predictions and never from data used for model training. AUC values were calculated from pooled out-of-fold predictions, with 95% confidence intervals estimated using the DeLong method. Sensitivity and specificity were calculated at the Youden-optimal threshold.

ROC/AUC analyses in the multi-disease setting were based on reduced binary contrasts rather than multiclass-derived metrics. That is, each ROC/AUC value was calculated for a specific pairwise disease comparison or one disease group versus a defined comparator group, rather than from a single multiclass ROC framework. Performance metrics included AUC with 95% confidence intervals, sensitivity, specificity, positive predictive value, negative predictive value, overall accuracy, balanced accuracy, and the optimal threshold. ROC curves and confusion matrix heatmaps were generated to visualize model performance. All analyses were performed using R (v4.4.1) and Python (v3.10), supported by in-house scripts and the caret, pROC, dplyr, tibble, and ggplot2 packages.

### 2.8 Ancillary clinical biomarker analyses

Ancillary analyses of protein-based biomarkers, including amyloid-*β* isoforms, total tau, and Mini-Mental State Examination (MMSE) scores, were plotted as boxplots using the raw values provided by the plasma supplier (BioIVT). Because proprietary normalization and diagnostic pipelines were not available, no additional normalization, transformation, or inferential testing was performed. Supplementary Figure 14 is therefore presented only as an orthogonal clinical context and qualitative comparison with cfDNA-derived results, and not as part of the primary cfDNA classifier analysis. Further analyses will be necessary to assess the degree of alignment between cfDNA-derived signals and orthogonal protein biomarkers, and to determine whether these modalities may serve complementary roles in disease characterization, stratification, and longitudinal monitoring.

## 3 Results

### 3.1 Whole-genome nanopore profiling identifies cell-type-specific methylation signatures

To establish our methylation reference atlas for cfDNA classification, we performed whole-genome methylation sequencing on genomic DNA from pooled human blood plasma and eight primary neuron and glial cell types using Wasatch BioLabs’ (WBL) Direct Whole Methylome Sequencing (dWMS) platform. This Oxford Nanopore Technologies (ONT)-based method captured native 5mC/5hmC methylation with single-molecule and CpG resolution.

Differential methylation analyses between pooled plasma and each neuronal and glial cell type identified millions of differentially methylated CpGs per comparison using a threshold of Δβ ≥ 0.8 (Supplementary Table 5), substantially exceeding the number identified via array. Among the primary neurons, cortical neurons exhibited the highest number of differentially methylated CpG sites (4.28 million), followed by dopaminergic neurons (1.75 million) and spinal motor neurons (1.54 million). Differential methylation analyses of glial cell types—astrocytes, microglia, and Schwann cells—relative to pooled blood plasma yielded between 1.24 and 1.43 million differentially methylated CpG sites. These analyses were included to illustrate the broader methylation features captured by whole-genome nanopore sequencing relative to array-based platforms.

**Figure 1:**
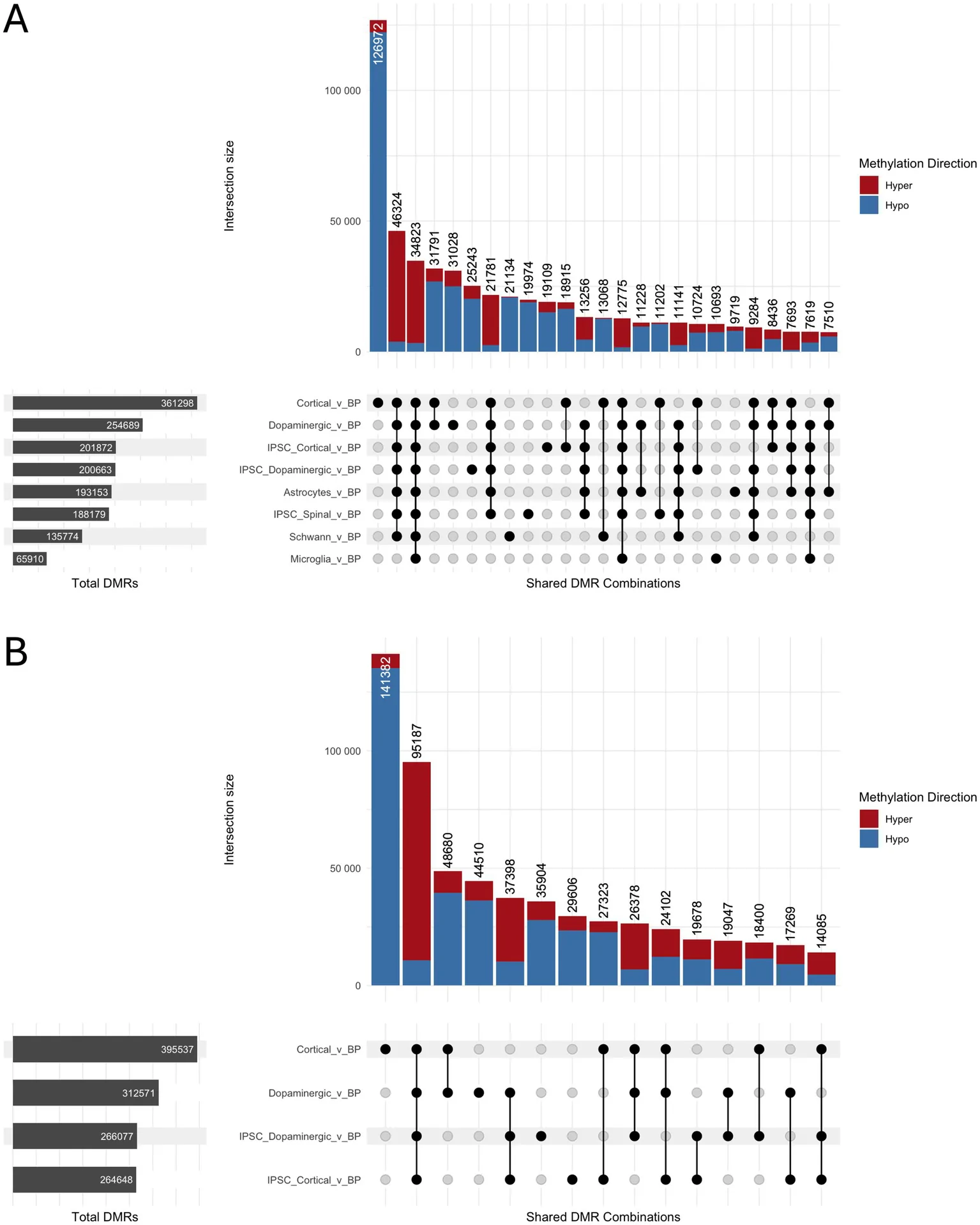
Differential methylation distinguishes neural cell types, primary versus iPSC-derived cells, and neurodegenerative disease states. Differential methylation analysis between each neural cell type and pooled human blood plasma identifies both shared and cell type-specific differentially methylated regions (DMRs), with primary cortical neurons exhibiting the largest number of unique DMRs (>120,000) **(A)**. Parallel analysis of plasma cfDNA from individuals with Alzheimer’s disease (AD), Parkinson’s disease (PD), amyotrophic lateral sclerosis (ALS), and controls reveals overlapping and disease-specific DMRs **(B)**. Across analyses, shared regions are predominantly hypermethylated (red), whereas increasingly cell- or disease-specific regions, hypomethylated (blue).

To assess cell type specificity, DMRs were compared across all neural populations, including both primary and iPSC-derived cell types. Thousands of overlapping and unique DMRs were identified per cell type. Figure 1A reports total, overlapping, and unique DMRs, with directional visualizations of methylation change (hypermethylation in red, hypomethylation in blue). Primary cortical neurons displayed 117,695 unique DMRs, primary dopaminergic neurons displayed 29,086 unique DMRs, primary spinal motor neurons displayed 89,648 unique DMRs (Figure 1A). Unique, non-redundant, cell type-specific DMRs for these cell types were used for downstream classifier development.

Comparing DMRs between iPSC-derived neurons and blood plasma to their primary counterparts revealed substantial divergence. Only 28.3% of DMRs in iPSC-derived cortical neurons overlapped with primary cortical neurons, 38.1% with dopaminergic neurons, and 6.8% with spinal motor neurons (Figure 1B). Although the total number of DMRs was comparable, their content and genomic distribution differed widely.

### 3.2 In silico validation demonstrates high analytical accuracy and specificity of cfDNA classifiers

**Figure 2:**
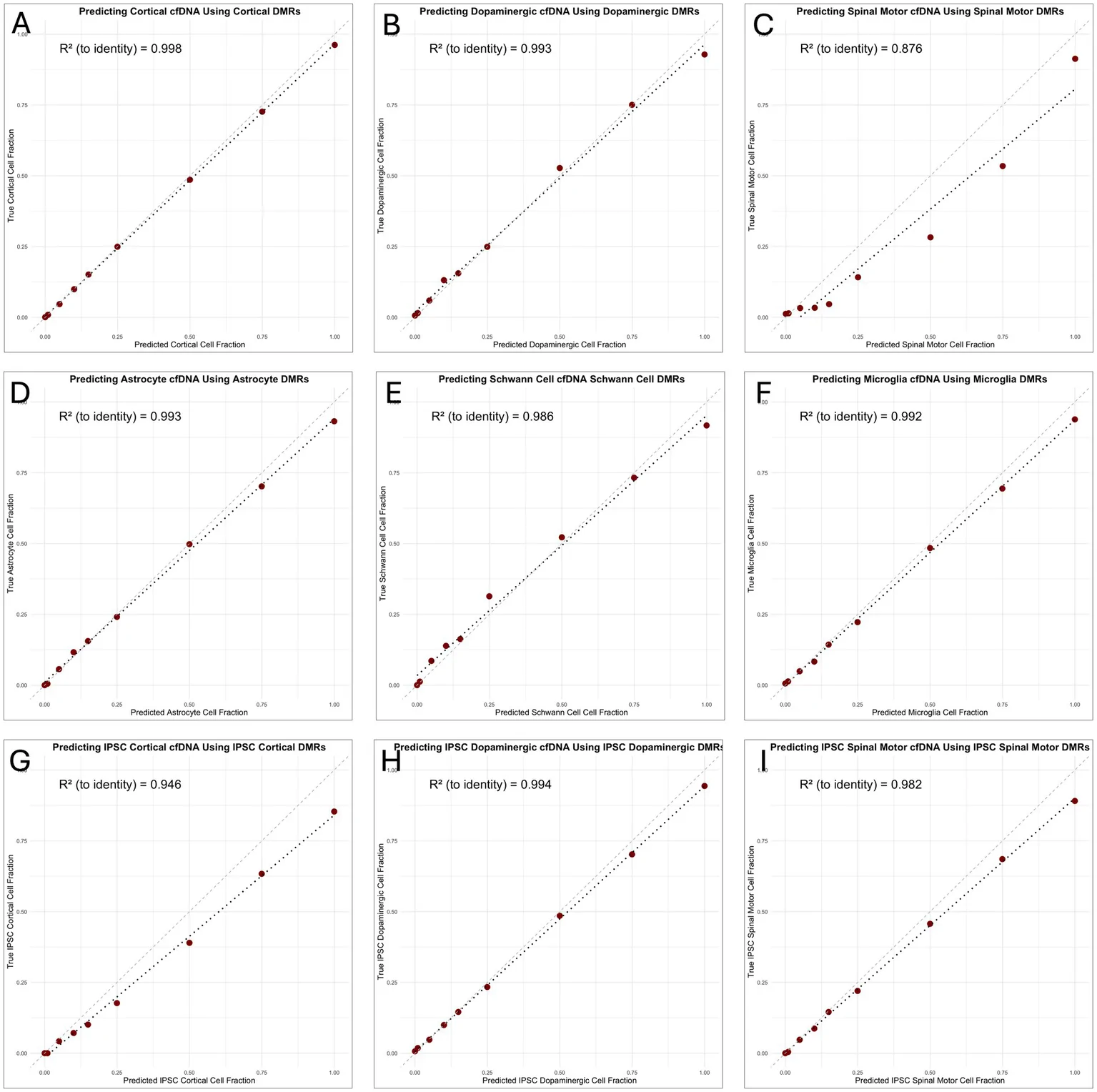
In silico validation demonstrates high accuracy and specificity of cell-type cfDNA classifiers. Correlation plots compare predicted versus true cell-type fractions across cfDNA-like in silico mixtures, in which fragmented cell-of-origin-derived reads were spiked into a background of human blood plasma reads. Each panel summarizes classifier performance for primary neuronal and glial cell types–primary cortical **(A)**, dopaminergic **(B)**, and spinal motor neurons **(C)**, astrocytes **(D)**, Schwann cells **(E)**, and microglia **(F)**, as well as iPSC-derived neurons, including cortical **(G)**, dopaminergic **(H)**, and spinal motor neurons **(I)**. Strong linearity and high concordance (R^2^ values) are observed when classifiers are applied to cfDNA from their matched cell of origin. Each point represents a single in silico dilution (~1,000,000 total reads), with nine fractional compositions (0–100% cell-type-derived reads) generated per cell type (Supplementary Table 4).

To assess the analytical accuracy and specificity of our DMR-based classifiers, we generated *in silico* cfDNA-like gDNA mixtures containing defined proportions of cell-type-specific reads in a plasma background and applied the classifiers. To better approximate native cfDNA properties, we computationally fragmented genomic DNA reads from purified cells, which were initially longer than plasma-derived cfDNA. Resultant fragment distributions more closely resembled circulating cfDNA (Supplementary Figure 1). *In silico* experiments of individual cell types also demonstrated that 5 predictive CpGs per molecule were sufficient for accurate read-level classification, supporting the windowing threshold used for classifier development (Figure 2).

The cortical neuron classifier showed the strongest performance, with strong agreement between predicted and measured cfDNA percentages across the dilution series (R^2^ = 0.998; Figure 2A). Similarly, high performance was observed for primary dopaminergic neurons (R^2^ to identity = 0.993; Figure 2B), astrocytes (R^2^ = 0.993; Figure 2D), Schwann cells (R^2^ to identity = 0.986; Figure 2E), and microglia (R^2^ to identity = 0.992; Figure 2F), each achieving R^2^ to identity values above 0.98.

Strong linearity was also observed across iPSC-derived *in silico* mixtures, including iPSC-derived cortical neurons (R^2^ to identity = 0.946; Figure 2G), dopaminergic neurons (R^2^ to identity = 0.994), and spinal motor neurons (R^2^ to identity = 0.982; Figure 2I). In contrast, primary spinal motor neuron-derived fractions and models developed from iPSC-derived neurons exhibited substantially reduced performance (R^2^ to identity = 0.876).

To assess cell type specificity, we also applied each classifier to *in silico* datasets from other cell types. In all cases, slopes for non-target cell types were near zero, indicating high model specificity for each cell type (Supplementary Figures 3–11).

### 3.3 Clinical proof of concept for cfDNA classifiers in patient plasma

#### 3.3.1 Neuron-specific cfDNA elevations distinguish Alzheimer’s, Parkinson’s, and ALS samples from controls

**Figure 3:**
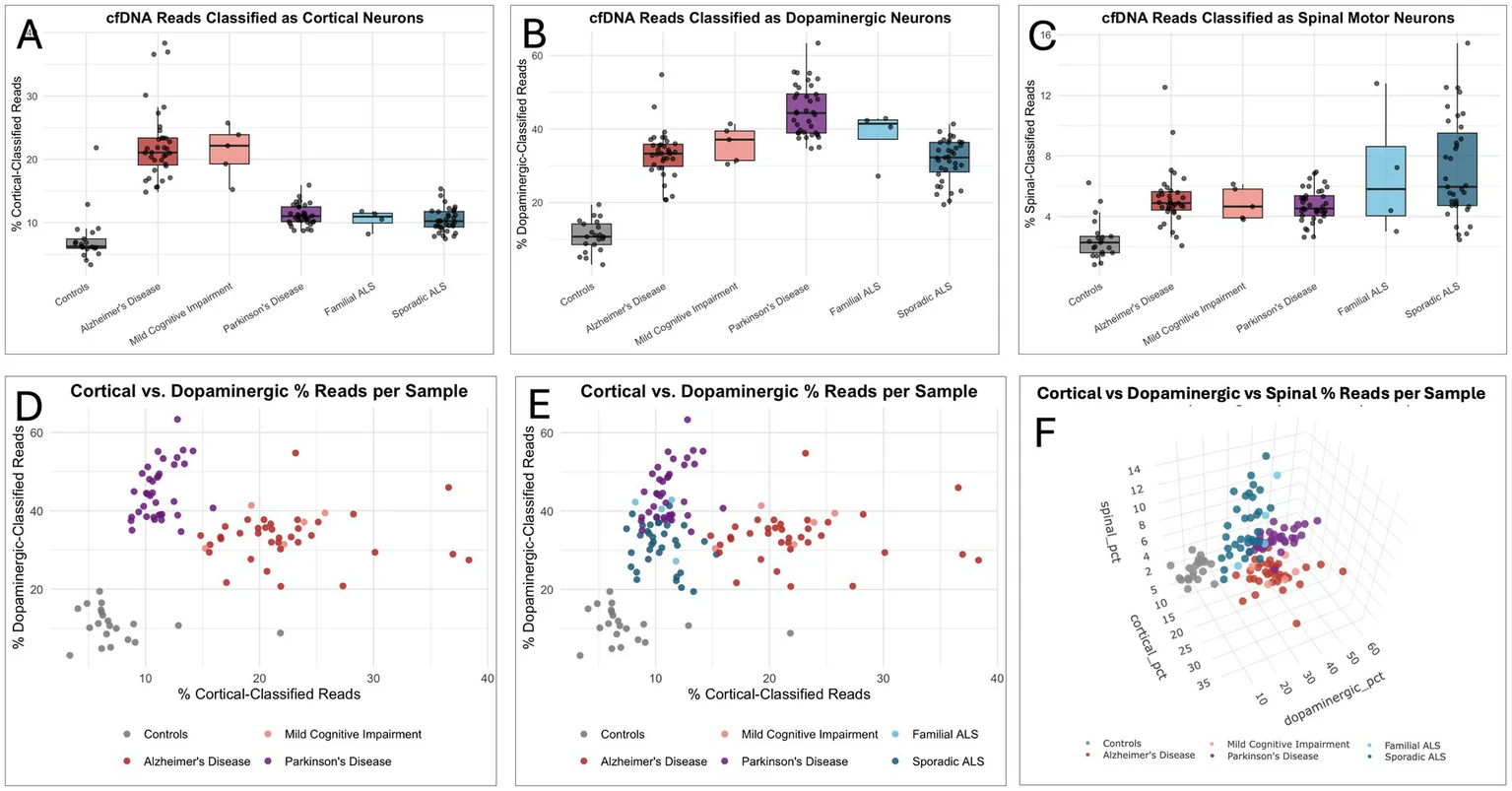
Neuron-like cfDNA classification aligns with selective neurodegeneration across diseases. Cell-of-origin classifiers for cortical **(A)**, dopaminergic **(B)**, and spinal motor neurons **(C)** were applied to cfDNA sequencing data from blood plasma of individuals with Alzheimer’s disease (AD, *n* = 35), mild cognitive impairment (MCI, *n* = 5), Parkinson’s disease (PD, *n* = 37), and amyotrophic lateral sclerosis (ALS, *n* = 39). Each classifier showed elevated neuron-derived cfDNA in its corresponding disease. Bivariate plots of cortical versus dopaminergic cfDNA fractions illustrate separation of AD and PD without **(D)** and with **(E)** inclusion of ALS. A three-dimensional plot integrating cortical (X-axis), dopaminergic (Y-axis), and spinal motor neuron (Z-axis) cfDNA further visualizes separation of disease states **(F)**.

After establishing classifier performance, we evaluated their clinical relevance by applying the primary neuron classifiers to the filtered plasma cohort of 137 samples from healthy controls and individuals with MCI, AD, PD, or ALS. Demographic characteristics of the final analytic cohort are summarized in Supplementary Table 3. Pooled cases were significantly older than controls (65.9 ± 10.4 vs. 58.2 ± 8.6 years; *p* = 0.001), whereas sex and ethnicity distributions did not differ significantly between groups (*p* = 0.2). The cfDNA analyses revealed significant disease-associated elevations in cfDNA signals aligned with selectively vulnerable neuronal populations, supporting the diagnostic potential of neuron-informed cfDNA methylation profiling (Figure 3).

Cortical neuron-associated cfDNA was significantly elevated in the combined AD+MCI group (22.27%, SD = 5.59%, *n* = 40) compared to controls (7.42%, SD = 3.86%, *n* = 21; BH-adjusted *p* = 2.40e-09), and also compared to the combined comparison group of controls, PD, and ALS (10.06%, SD = 2.72%, *n* = 97; BH-adjusted *p* = 8.64e-19; Figures 3A,D). Dopaminergic neuron-associated cfDNA was significantly elevated in PD (45.01%, SD = 6.95%, *n* = 37) relative to controls (10.94%, SD = 4.24%, *n* = 21; BH-adjusted *p* = 6.88e-10), and relative to the combined comparison group of controls, AD, MCI, and ALS (28.25%, SD = 10.72%, *n* = 100; BH-adjusted *p* = 8.55e-15; Figures 3B,E). Spinal motor neuron-associated cfDNA was significantly elevated in ALS (7.11%, SD = 3.37%, n = 39) compared to controls (2.48%, SD = 1.34%, *n* = 21; BH-adjusted *p* = 4.31e-08), and relative to combined controls, AD, MCI, and PD (4.42%, SD = 1.77%, *n* = 98; BH-adjusted *p* = 1.23e-05; Figures 3C,F).

By contrast, classifiers for astrocytes, Schwann cells, microglia, and iPSC-derived neurons showed no consistent disease-associated changes in patient plasma relative to controls (Supplementary Figure 14).

Together, these results are consistent with our qualitative observation that iPSC-derived neuron methylation profiles differ substantially from those of primary neurons. More broadly, they indicate that primary-cell-based classifier frameworks may capture disease-relevant signal not reproduced by iPSC-derived reference models, reinforcing the value of cell-type-resolved cfDNA methylation profiling for noninvasive investigation of neurodegeneration.

#### 3.3.2 Integrating multiple neuronal cfDNA signals enhances disease prediction

To further improve disease prediction accuracy, we applied cortical, dopaminergic, and spinal motor neuron classifiers in an integrated model. The integrated model was tested in both binary (e.g., AD vs. controls, PD vs. controls, ALS vs. controls) and multi-disease (e.g., AD vs. all other diseases and controls) contexts via ROC analysis, which enabled side-by-side comparisons of univariate and multivariate models.

**Figure 4:**
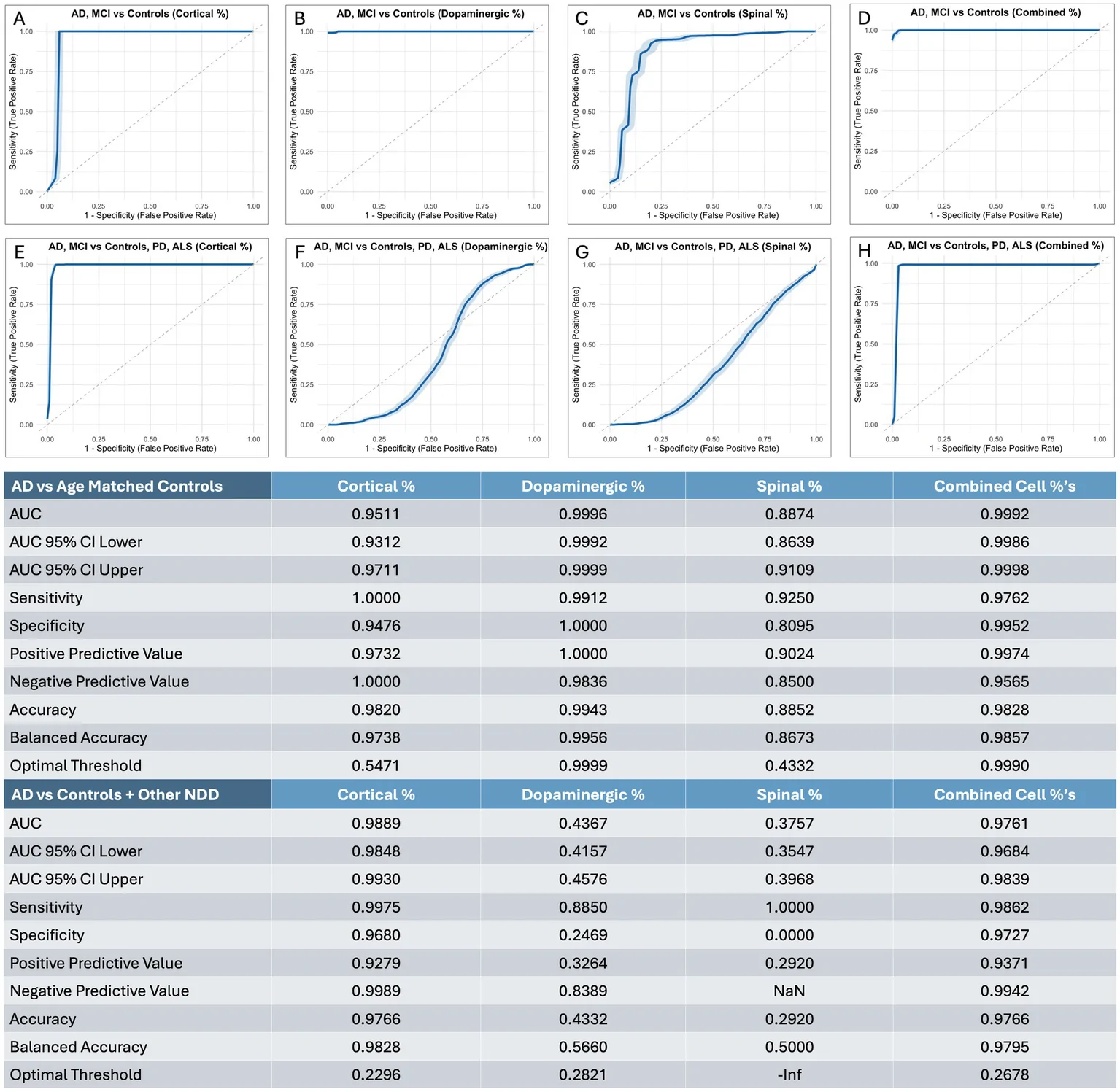
Multivariate primary neuron cfDNA classifiers improve discrimination of Alzheimer’s disease. Receiver operating characteristic (ROC) curves evaluate the performance of classifiers derived from primary neuronal reference profiles applied to blood plasma from healthy controls (*n* = 21) and individuals with Alzheimer’s disease (AD, *n* = 35), mild cognitive impairment (MCI, *n* = 5), and other neurodegenerative diseases (NDDs). Classifiers based on cortical **(A,E)**, dopaminergic **(B,F)**, and spinal motor neuron reference profiles **(C,G)** were evaluated individually and in combination as a multivariate model **(D,H)**. Panels compare classification of AD and MCI versus controls **(A-D)** and versus controls plus other NDDs, including Parkinson’s disease (*n* = 37) and amyotrophic lateral sclerosis (*n* = 39; **E–H**). Integration of multiple neuron-aligned cfDNA methylation signals improved discriminative performance in multi-disease comparisons, supporting the added value of multivariate cfDNA methylation profiling in more complex clinical contexts.

The multivariate classifiers generally yielded AUC values comparable to or higher than those of the univariate counterparts, generally showing improved sensitivity, specificity, and overall accuracy. For the binary AD vs. control analysis, the individual cortical neuron classifier produced an AUC = 0.9511 (95% CI: 0.9312–0.9711), with 1.0000 sensitivity, 0.9476 specificity, corresponding positive and negative predictive values (PPV and NPV) of 0.9732 and 1.0000, respectively, and balanced accuracy of 0.9738 at the optimal classification threshold of 0.5471 (Figure 4). The multi-cell model that integrated all 3 neurons maintained performance, with AUC = 0.9992 (95% CI: 0.9986–0.9998), with 0.9762 sensitivity, 0.9952 specificity, corresponding positive and negative predictive values (PPV and NPV) of 0.9974 and 0.9565, respectively, and balanced accuracy of 0.9857 at the optimal classification threshold of 0.9990.

**Figure 5:**
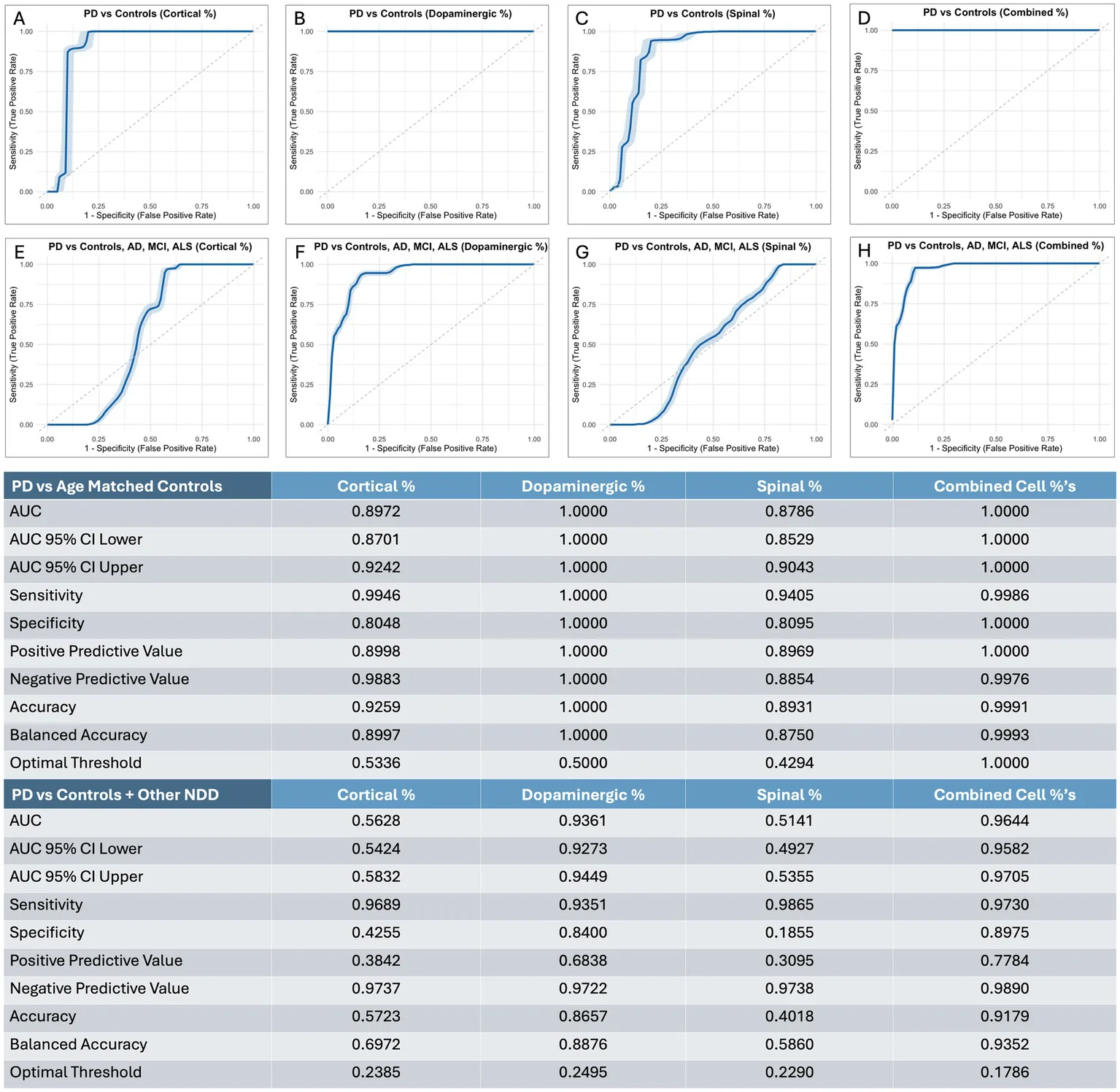
Multivariate primary neuron cfDNA classifiers improve discrimination of Parkinson’s disease. Receiver operating characteristic (ROC) curves evaluate the performance of classifiers derived from primary neuronal reference profiles applied to blood plasma from healthy controls (*n* = 21) and individuals with Parkinson’s disease (PD, *n* = 37) and other neurodegenerative diseases (NDDs). Classifiers based on cortical **(A,E)**, dopaminergic **(B,F)**, and spinal motor neuron reference profiles **(C,G)** were assessed individually and in combination as a multivariate model **(D,H)**. Panels compare classification of PD versus controls **(A–D)** and versus controls plus other NDDs, including Alzheimer’s disease (*n* = 35) and amyotrophic lateral sclerosis (*n* = 39; **E–H**). Integration of multiple neuron-aligned cfDNA methylation signals improved discriminative performance in multi-disease comparisons, supporting the added value of multivariate cfDNA methylation profiling in more complex clinical settings.

To further demonstrate the added value of integrating multiple cell-type signatures into one model, we compared the performance of the combined classifier to the corresponding univariate models across PD and ALS cases. In the PD comparisons (Figure 5), while both the dopaminergic and combined models achieved similar separation in the PD versus control comparisons (AUC = 1.0000), only the combined model maintained strong performance when distinguishing PD from ALS and AD. In contrast, cortical and spinal motor classifiers, which performed well in PD vs. control settings (AUC = 0.8972 and 0.8786, respectively), showed decline in the three-class setting (AUC = 0.5628 and 0.5141). The combined model effectively aggregated discriminative signals across relevant and partially overlapping cell types, achieving AUC = 0.9644 (95% CI: 0.9582–0.9705), 0.9730 sensitivity, 0.8975 specificity, PPV and NPV of 0.7784 and 0.9890, respectively, and balanced accuracy of 0.9352 at the optimal threshold of 0.1786.

**Figure 6:**
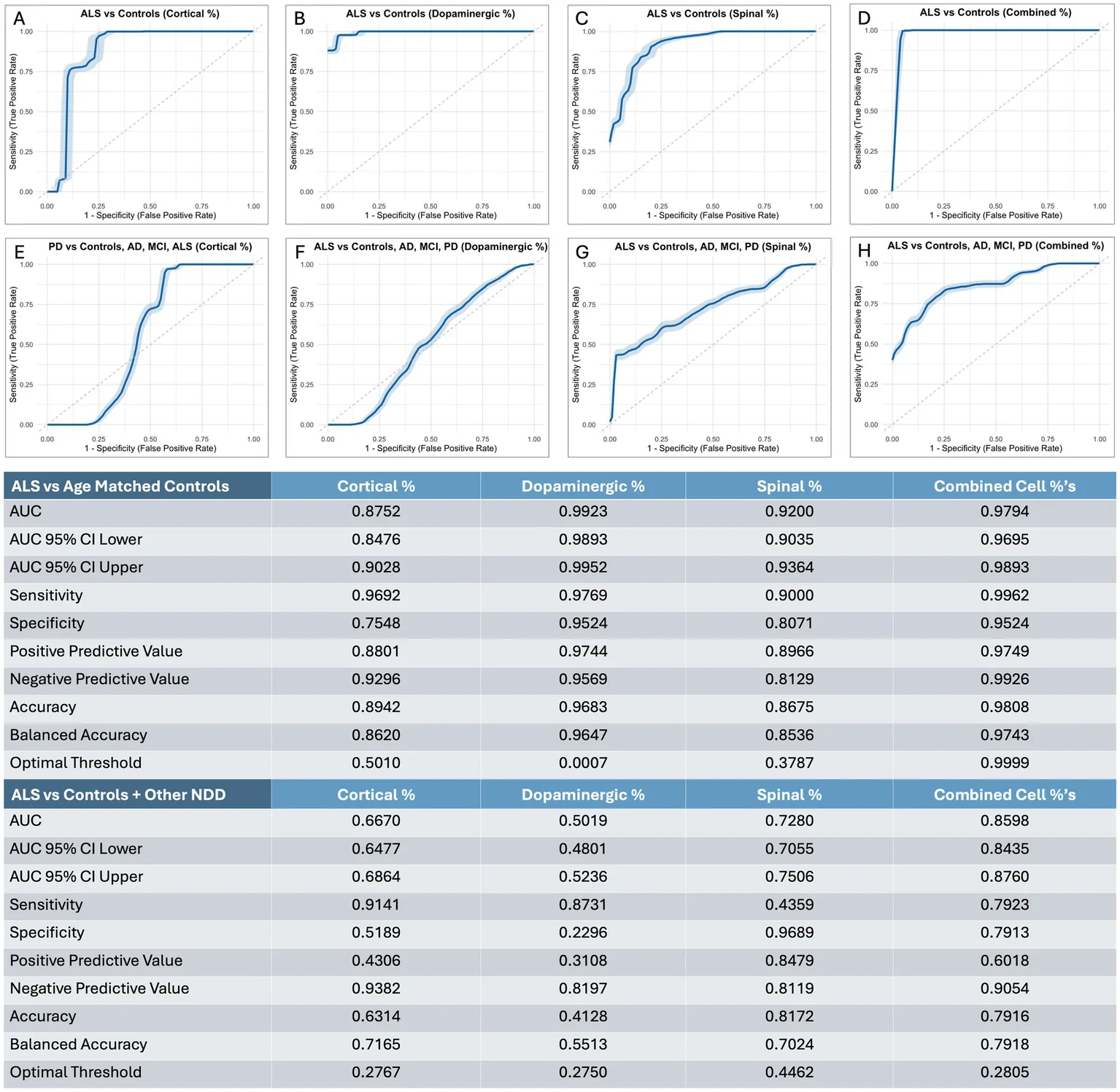
Multivariate primary neuron cfDNA classifiers improve discrimination of amyotrophic lateral sclerosis. Receiver operating characteristic (ROC) curves evaluate the performance of classifiers derived from primary neuronal reference profiles applied to blood plasma from healthy controls (*n* = 21) and individuals with amyotrophic lateral sclerosis (ALS, *n* = 39) and other neurodegenerative diseases (NDDs). Classifiers based on cortical **(A,E)**, dopaminergic **(B,F)**, and spinal motor neuron reference profiles **(C,G)** were assessed individually and in combination as a multivariate model **(D,H)**. Panels compare classification of ALS versus controls **(A–D)** and versus controls plus other NDDs, including Alzheimer’s disease (*n* = 35) and Parkinson’s disease (*n* = 37; **E–H**). Integration of multiple neuron-aligned cfDNA methylation signals improved discriminative performance in multi-disease comparisons, supporting the added value of multivariate cfDNA methylation profiling in more complex clinical settings.

A similar pattern was observed for ALS (Figure 6). The spinal motor classifier alone showed strong binary discrimination (AUC = 0.9200), but performance declined in the multi-disease setting (AUC = 0.7280). Other univariate classifiers also performed well in ALS versus control comparisons (cortical: 0.8752; dopaminergic: 0.9923), but similarly lost discriminative power in the multi-disease context (AUCs = 0.6670 and 0.5019, respectively). By contrast, the combined ALS model maintained stronger discrimination (AUC = 0.8598), highlighting the value of integrating complementary cell-type signals to improve disease specificity in more complex clinical settings.

These cell type-specific disease predictions were not recapitulated when iPSC-derived models were applied, whether individually or in combined multi-cell formats, and in both binary and multi-disease settings (Supplementary Figures 13–15). Overall, these results suggest that although neuron-aligned classifiers may capture disease-relevant signal, integrating cfDNA methylation features across neuronal populations improves discrimination and specificity in more clinically realistic settings, where contributions from multiple cell types and multiple disease states are likely to coexist.

## 4 Discussion

The data presented here support our previous findings in AD (30) and provide proof of concept evidence that native nanopore sequencing can support reference atlas generation and downstream cfDNA cell-of-origin classifier development for neurodegenerative disease. Across AD, PD, and ALS, elevated circulating cfDNA fragments exhibited methylation patterns similar to reference profiles from selectively vulnerable neuronal populations, with cortical neuron-like signatures elevated in AD and progressive MCI, dopaminergic neuron-like signatures in PD, and spinal motor neuron-like signatures in ALS. These signals supported strong binary disease-versus-control discrimination (AUCs > 0.97) and remained informative in more clinically relevant multi-disease comparisons (AUCs > 0.85; Figures 4–6).

Although the current reference atlas is currently limited in scope, these findings suggest the feasibility of applying native cfDNA methylation sequencing for detecting disease-associated, neuron-linked neurodegeneration signals from peripheral blood plasma, extending prior work in neurodegeneration and other neurological conditions (1, 4, 24, 26, 31).

Cell-of-origin deconvolution from bulk tissues and mixed biological matrices is well established. Although foundational studies have largely relied on methylation arrays or bisulfite-based sequencing approaches (17–19, 51, 52), they have enabled inference of tissue composition across a wide range of biological contexts. In healthy adults, these studies have estimated that circulating cfDNA is predominantly hematopoietic in origin, with contributions from white blood cells (55%), erythrocyte progenitors (30%), vascular endothelial cells (10%), and hepatocytes (1%) (17). Applying this framework to cell-free DNA in neurodegenerative disease, however, presents additional challenges.

Neuron-derived cfDNA is expected to comprise only about 2% of total circulating DNA (17). Bisulfite conversion induces DNA damage and fragmentation, further reducing already limited input material, while PCR amplification introduces bias and reduces quantitative fidelity (27–29). Array-based approaches additionally restrict analysis to predefined CpG sites and regions, limiting coverage and the ability to detect cell-specific patterns in sparse or heterogeneous samples. Native nanopore sequencing offers a promising solution by directly profiling endogenous methylation without bisulfite conversion or amplification, preserving native cfDNA molecules while reducing associated biases and enabling genome-wide methylation discovery (32). This is especially valuable for brain cell-type deconvolution, where informative CpGs may fall outside standard array content and overlapping methylation programs between related neuronal populations must be progressively refined. Nanopore sequencing also enables flexible expansion of the reference atlas as additional cell types become available, consistent with emerging applications in native methylation profiling and low-coverage cfDNA liquid biopsy workflows (33–35).

Prior cfDNA methylation studies, including our earlier targeted assay in AD and MCI, have been limited by target locus restriction and the constraints of bisulfite-based profiling (30). More broadly, prior cfDNA methylation studies have largely relied on array- or bisulfite-based methods that interrogate only a subset of CpG loci and capture only a small fraction of the methylome (53). Although the human genome contains approximately 30 million CpG sites, standard arrays survey approximately 900,000, or approximately 3%, with strong bias toward CpG islands and other CpG-rich regions (18, 53, 54). Here, whole-genome nanopore sequencing of purified primary neural populations and plasma cfDNA expanded CpG coverage beyond methylation arrays (Supplementary Table 5), enabling broader capture and refinement of methylation signatures for cortical, dopaminergic, and spinal motor neurons.

The human brain is arguably the most complex organ in the human body due to its cellular composition and diversity (55). Changes in the relative proportions of neurons, glia, and other neural cell types have been implicated in neurodegenerative, psychiatric, and aging-related processes. For example, reduced volume and number of neurons of Broadmann’s area 17 have been associated with schizophrenia (56). Cell-counting studies have identified changes in the density and size of both neurons and glia in a number of frontolimbic regions, including the dorsolateral prefrontal, orbitofrontal, and anterior cingulate cortex, and the amygdala and hippocampus in clinical mood disorders like depression (57). However, accurate measurement of brain cellular composition remains challenging due to neuronal heterogeneity within and across regions, and technical limitations of available methods (58, 59). Neuron nuclei markers, such as NeuN, identify neurons but cannot distinguish individual neuron types (60).

Direct interrogation of DNA methylation offers a scalable approach for deconvoluting human brain cell-type diversity. In contrast to single-nucleus methods, methylation-based deconvolution from biological matrices like blood plasma does not require specialized sample processing, is more cost-effective, and is readily adaptable to large cohort studies (2). Hierarchical deconvolution frameworks have been used to resolve major brain cell populations, including GABAergic and glutamatergic neurons, astrocytes, microglia, oligodendrocytes, endothelial cells, and stromal cells, although these studies have largely relied on publicly available array-based datasets for marker selection (2). Against this backdrop, the present work extends the existing landscape by testing whether native nanopore sequencing can be used to generate a primary human brain methylation reference atlas and apply it to cfDNA-based cell-of-origin deconvolution.

Here, we included cortical, dopaminergic, and spinal motor neurons due to their associations with the neurodegenerative diseases of interest (61–64), as well as three glial cell types. Although we used a reference-based classifier framework (51), which has been shown to outperform unsupervised machine learning approaches (65, 66), we acknowledge our atlas is limited relative to the cellular diversity present in the brain. Pooled plasma served as a practical proxy for the predominant blood-derived contributors to circulating cfDNA, with major contributions from white blood cells and erythrocyte progenitors in healthy individuals (17). While we determined this was sufficient for proof-of-concept classifier development, we acknowledge it does not capture the full range of potentially confounding peripheral or CNS-derived cfDNA sources. This limited atlas likely contributed to the comparatively high neuron-derived cfDNA fractions estimated here, which exceed prior reports (17, 67–69). These estimates likely reflect residual overlap in methylation signals, incomplete exclusion of non-neuronal sources, and capture of broader cell death-associated patterns rather than a fully neuron-restricted signal. Additionally, because reads with indeterminate methylation profiles were excluded from our analyses, these results suggest that many omitted reads were likely non-neuronal, potentially inflating the apparent neuronal fraction. It is also possible that neuron-derived cfDNA has a fragment-size profile preferentially retained or enriched by our workflow. Some studies suggest that necrosis produces longer cfDNA fragments than apoptotic pathways (11, 70, 71) and may, themselves, function as a biomarker of many human diseases including ischemic injury and neurodegenerative disease. Necroptosis, a genetically regulated form of necrotic cell death, has been shown to link aging, inflammation, and cognition (72). Future studies will expand the atlas to include purified hematopoietic populations, as well as endothelial, skeletal muscle, hepatocytic, and other peripheral reference cell types, and more explicitly evaluate fragment-size effects to improve specificity and reduce shared or non-specific cell death-associated signals.

While our neuronal classifiers performed well in disease-versus-control comparisons, we observed weaker performance in multi-disease comparisons, unless features were combined into multivariate models, yielding a rescuing effect (Figures 4–6). This pattern may suggest that integrating partially distinct neuronal signatures captures complementary aspects of disease-associated neurodegeneration that are not fully represented by any single classifier. However, it may also indicate that the classifiers are detecting shared, non-specific signals related to disease state more broadly, or other correlated biological and technical variables, rather than purely cell-type-resolved degeneration. Distinguishing among these possibilities will require validation in larger cohorts with sufficient power to model covariates such as age, sex, batch, and other potential contributors to non-cell-type-specific signal (73–75). Thus, while the multivariate results are encouraging, they should be interpreted within the proof-of-concept scope of the present study.

As an exploratory alternative to primary cells, we evaluated iPSC-derived neurons as a more accessible substrate for classifier training. However, classifiers trained on iPSC-derived neurons failed in both *in silico* admixture analyses and clinical plasma samples from patients with AD, PD, and ALS. These results were consistent with our qualitative observation that iPSC-derived neuronal methylomes differed substantially from their primary counterparts, suggesting that key cell-of-origin methylation features were not faithfully recapitulated. Although this finding could also suggest the classifiers’ sensitivity to specific reference methylomes used for training and therefore limit generalizability, the preserved performance of primary-cell-trained classifiers in independent patient plasma samples suggested that inadequate biological fidelity of the iPSC-derived references is more likely. This interpretation is supported by other work demonstrating persistent epigenetic memory and incomplete epigenetic reprogramming in iPSC-derived cells, including unresolved aberrations that persist even after differentiation (76–79).

Looking ahead, the availability of primary tissue poses a practical challenge for sourcing and expanding our primary neuron atlas. This was evident for spinal motor neurons, which are rarely available from commercial sources and required direct isolation from postmortem human spinal cord tissue using an optimized Percoll gradient protocol (40). Although this approach is likely less specific than FACS- or FANS-based isolation methods used in recent methylation atlas studies (17), the selective elevation of spinal motor neuron-associated cfDNA in ALS suggested core disease-relevant methylation features were captured. More broadly, the field will benefit from continued development of primary human reference resources, including brain-region-specific and subtype-resolved methylation atlases generated across diverse donors and demographic backgrounds. Recent nanopore-based work using sorted nuclei populations from human cortex further supports the feasibility of generating high-coverage, cell-type-resolved brain methylation references using ONT sequencing, with strong concordance to EPIC array and EM-seq data (80).

The present study should therefore be understood as feasibility work at two levels. First, we demonstrate that native nanopore sequencing can generate a primary human brain methylation atlas suitable for classifier training. Second, we demonstrate that native cfDNA nanopore methylation sequencing can recover disease-relevant, neuron-associated signals from peripheral blood. With this feasibility framing, the current work should be interpreted with several considerations and limitations. The current reference atlas is not comprehensive, the patient cohorts are small to modest in size, and blood plasma data are inherently cross-sectional, limiting longitudinal insight. In addition, pooled cases and controls differed in age in the final analytic cohort, reflecting both the use of a shared control group across multiple disease cohorts with different age distributions and the practical constraints of sourcing samples from commercial biobank vendors. PCA analysis of plasma methylation features did not identify age as a dominant source of global variation. Although this does not entirely exclude the possibility of age-related confounding, this finding indicates that the observed classifier-associated disease signal is not explained by an obvious global age effect alone (Supplementary Figure 1; Supplementary Table 3). Other potential sources of technical and biological confounding, including batch effects related to collection history, sample handling, and sequencing batch (73–75), remain possible and will be addressed in future studies.

Despite these limitations, the clinical potential of cfDNA in neurodegeneration is compelling. Neurodegenerative diseases often unfold over long prodromal periods, with neuronal loss beginning years before the onset of overt clinical symptoms (81–85). Because cfDNA has a short half-life of minutes to hours, it may provide a more temporally resolved readout of active cell death than protein biomarkers that reflect slower-changing processes over days to weeks (86). In this context, neuron-associated cfDNA could complement existing blood-based protein biomarkers by providing information not only about whether neurodegeneration is occurring, but also about which neuronal systems are most affected. Notably, all individuals with MCI in our cohort who later progressed to AD exhibited elevated cortical cfDNA at the time of sampling, suggesting potential early disease relevance that warrants prospective validation. However, the small number of MCI samples (n = 5) and the limited demographic diversity of the cohort, constrain generalizability and should be noted.

This study contributes to the broader landscape of brain-derived cfDNA research by demonstrating native nanopore sequencing as a flexible platform for future atlas and classifier development. As additional neuronal, glial, and peripheral cell types are incorporated, this framework could enable finer discrimination of region- and cell-type-specific neurodegenerative processes. The same flexibility may also support extension to other neurological conditions characterized by selective cellular vulnerability, including brain cancers, multiple sclerosis, traumatic brain injury, stroke, epilepsy, and neuropsychiatric disease. For example, elevated levels of neuron-, astrocyte-, oligodendrocyte-, and whole-brain-derived cfDNA have been reported in the plasma of schizophrenia patients during a first psychotic episode (87). Altered neuronal-to-glial cfDNA ratios have also been shown to distinguish primary psychiatric disease from behavioral-variant frontotemporal dementia in blood serum (26).

Parallel work in oncology supports the broader applicability of circulating DNA-based approaches. Early studies suggested that brain tumor-derived signals may be difficult to detect in peripheral blood due to obstacles such as the blood–brain barrier. However, foundational work demonstrated that circulating tumor DNA is detectable across multiple malignancies, albeit at substantially lower frequencies in primary brain tumors, with <10% of glioma patients showing detectable ctDNA in plasma (88). These findings supported both the feasibility and highlighted limitations of plasma-based ctDNA detection in neuro-oncology, specifically the challenge of low circulating tumor DNA abundance in brain-derived disease. Despite this, subsequent studies have shown that plasma cfDNA methylation signatures can accurately detect and discriminate intracranial tumor types that can be challenging to identify via standard imaging approaches (89). Methylation-based cfDNA approaches have also been used to develop and validate predictive models for early detection of brain metastases, leveraging genome-wide methylation features to classify patient risk for central nervous system involvement in lung adenocarcinoma, the most common source of brain metastases (90).

As native-read sequencing technologies become increasingly practical for translational research (35, 36, 91–97), direct methylation profiling without chemical conversion or amplification may offer an important advance for liquid biopsy applications in settings where the biological signal is sparse and input material is limited. In this context, direct analysis of native DNA has been described as the “holy grail” of methylation sequencing (93).

In summary, the data presented here support the feasibility of using native nanopore sequencing for both the generation of a brain methylation reference atlas and cfDNA cell-of-origin analysis in neurodegenerative disease. Although the current study is a proof-of-concept and is limited by the breadth of the atlas, cohort size, and incomplete control of potential confounders, it demonstrates that native cfDNA methylation sequencing can recover neuron-associated signals that largely align with selectively vulnerable populations in AD, PD, and ALS. Additional validation, including repeated cross-validation, permutation testing, and evaluation in independent external cohorts, will be necessary to determine the extent to which the observed discrimination reflects robust biological signal versus cohort-specific effects. With continued atlas expansion, larger longitudinal cohorts, and more formal benchmarking against existing deconvolution approaches, nanopore-based cfDNA profiling may provide a scalable and biologically informative liquid biopsy framework for neurodegeneration.

## Data Availability

The original contributions presented in the study are publicly available. This data can be found here: Data has been made available NCBI’s Sequence Read Archive (SRA) under the project name ‘Circulating neuron-derived cfDNA for blood-based detection of Alzheimer’s and other neurodegenerative conditions’ and under the BioProject ID PRJNA1503594.

## Footnotes

1
https://github.com/samtools

2
https://github.com/nanoporetech/dorado

3
https://github.com/nanoporetech/modkit

4
https://github.com/Wasatch-Biolabs-Bfx/MethylSeqR
