# Supplementary material for: Circulating neuron-derived cfDNA for blood-based detection of Alzheimer’s and other neurodegenerative conditions
Source: Front Neurol. 2026 Aug 6;17:1822479. doi: 10.3389/fneur.2026.1822479 (PMC13491437; doi:10.3389/fneur.2026.1822479)
Supplement: Supplementary file 1 [file Data_Sheet_1.PDF]

# *Supplementary Material*

## 1 Supplementary Tables

### 1.1 Supplementary Table 1. Cell types used for model training and analytical validation, including cell origin (primary or iPSC-derived), vendor, and catalog numbers.

| *Cell Type* | *Origin* | *Vendor* | *Catalog #* |
|---|---|---|---|
| *Human Cortical Neurons* | Primary | Creative BioArray | CSC-C1514 |
| *Human Cortical Neurons* | iPSC-Derived | Creative BioArray | CSC-00834L1 |
| *Human Dopaminergic Neurons* | Primary | Creative BioArray | CSC-C30012 |
| *Human Dopaminergic Neurons* | iPSC-Derived | Creative BioArray | CSC-00842L |
| *Human Spinal Motor Neurons* | iPSC-Derived | Accegen | ABC-SC112G |
| *Human Astrocytes* | Primary | Creative BioArray | CSC-7836W |
| *Human Schwann Cells* | Primary | Creative BioArray | CSC-7836W |
| *Human Microglia* | Primary | Creative BioArray | CSC-C1527 |

1.2 **Supplementary Table 2. Allocation of human blood plasma samples for classifier training and testing after final cohort filtering.** A total of 137 blood plasma samples from healthy controls and individuals with mild cognitive impairment (MCI), Alzheimer's disease (AD), Parkinson's disease (PD), sporadic amyotrophic lateral sclerosis (sALS), and familial amyotrophic lateral sclerosis (fALS) were retained after filtering and split into training and testing sets using a 30% / 70% allocation.

| *Disease Group* | *Training (n)* | *Testing (n)* | *Total (n)* |
|---|---|---|---|
| *Control* | 6 | 15 | 21 |
| *MCI* | 2 | 3 | 5 |
| *AD* | 11 | 24 | 35 |
| *PD* | 11 | 26 | 37 |
| *sALS* | 11 | 24 | 35 |
| *fALS* | 1 | 3 | 4 |
| *Total* | 42 | 95 | 137 |

1.3 **Supplementary Table 3. Human plasma sample demographics for the final analytic cohort.** Demographic characteristics of healthy controls and combined disease cases are shown for the 137 plasma samples retained after filtering. Values are presented as mean ± SD for age and n (%) for categorical variables. P values reflect control-versus-case comparisons using Welch's two-sample t-test (unequal variances) for age and Fisher's exact test for sex and ethnicity.

| ***Characteristic*** | ***Group*** | | | ***P value*** |
|---|---|---|---|---|
| | **Overall**<br>N = 137 | **Control**<br>N = 21 | **Case**<br>N = 116 | |
| ***Age (years)*** | 64.7 ± 10.5 | 58.2 ± 8.6 | 65.9 ± 10.4 | 0.001 |
| ***Sex*** | | | | 0.2 |
| *Male* | 86 (63%) | 16 (76%) | 70 (60%) | |
| *Female* | 51 (37%) | 5 (24%) | 46 (40%) | |
| ***Ethnicity*** | | | | 0.2 |
| *African American* | 24 (18%) | 4 (19%) | 20 (17%) | |
| *Caucasian* | 87 (64%) | 11 (52%) | 76 (66%) | |
| *Hispanic* | 24 (18%) | 5 (24%) | 19 (16%) | |
| *Native American* | 1 (0.7%) | 1 (4.8%) | 0 (0%) | |
| *Vietnamese* | 1 (0.7%) | 0 (0%) | 1 (0.9%) | |

1.4 **Supplementary Table 4. Detailed breakdown of in silico dilution datasets, including the proportion and absolute number of cell-derived and plasma-derived reads in each mixture.** Genomic DNA from primary human neurons and glial cells was whole-genome nanopore sequenced, computationally fragmented to mimic cfDNA, and mixed with blood plasma-derived cfDNA reads at defined concentrations.

| | *Purified Cell Type %* | *Purified Cell Type Read Count* | *Blood Plasma %* | *Blood Plasma Read Count* |
|---|---|---|---|---|
| *Set 1* | 0.0% | 0 | 100.0% | 1000000 |
| *Set 2* | 0.1% | 1000 | 99.9% | 999000 |
| *Set 3* | 1.0% | 10000 | 99.0% | 990000 |
| *Set 4* | 10.0% | 100000 | 90.0% | 900000 |
| *Set 5* | 15.0% | 150000 | 85.0% | 850000 |
| *Set 6* | 25.0% | 250000 | 75.0% | 750000 |
| *Set 7* | 50.0% | 500000 | 50.0% | 500000 |
| *Set 8* | 75.0% | 750000 | 25.0% | 250000 |
| *Set 9* | 100.0% | 1000000 | 0.0% | 0 |

### 1.5 Supplementary Table 5. Predictive CpG's between the EPIC array and direct nanopore methylation sequencing for primary human brain cells.

| *Differentially Methylated Regions* | *dMS (28M+ CpG's)* | *EPIC Array (900k CpG's)* |
|---|---|---|
| *Cortical Neurons* | 4,276,222 | 9,199 |
| *Spinal Motor Neurons* | 1,534,893 | 5,030 |
| *Dopaminergic Neurons* | 1,753,954 | 2,223 |
| *Schwann Cells* | 1,239,734 | NA |
| *Microglia* | 1,328,016 | NA |
| *Astrocytes* | 1,431,593 | NA |

# 2 Supplementary Figures

## 2.1 Supplementary Figure 1. Principal component analysis of plasma-sample methylation features by flow cell, diagnostic group, sex, and age.

**Supplementary Figure 1. Principal component analysis of plasma-sample methylation features by flow cell, diagnostic group, sex, and age.** To assess whether major axes of variation were driven by technical or demographic variables, we performed PCA on all plasma samples and generated a scree plot showing the proportion of variance explained by each principal component (A), along with PCA plots colored by flow cell (B), sex (C) and diagnostic group, and (D) age. No single principal component showed dominant separation by diagnostic group (B–D), suggesting that disease-associated signal is not explained by an global batch or demographic effect alone.

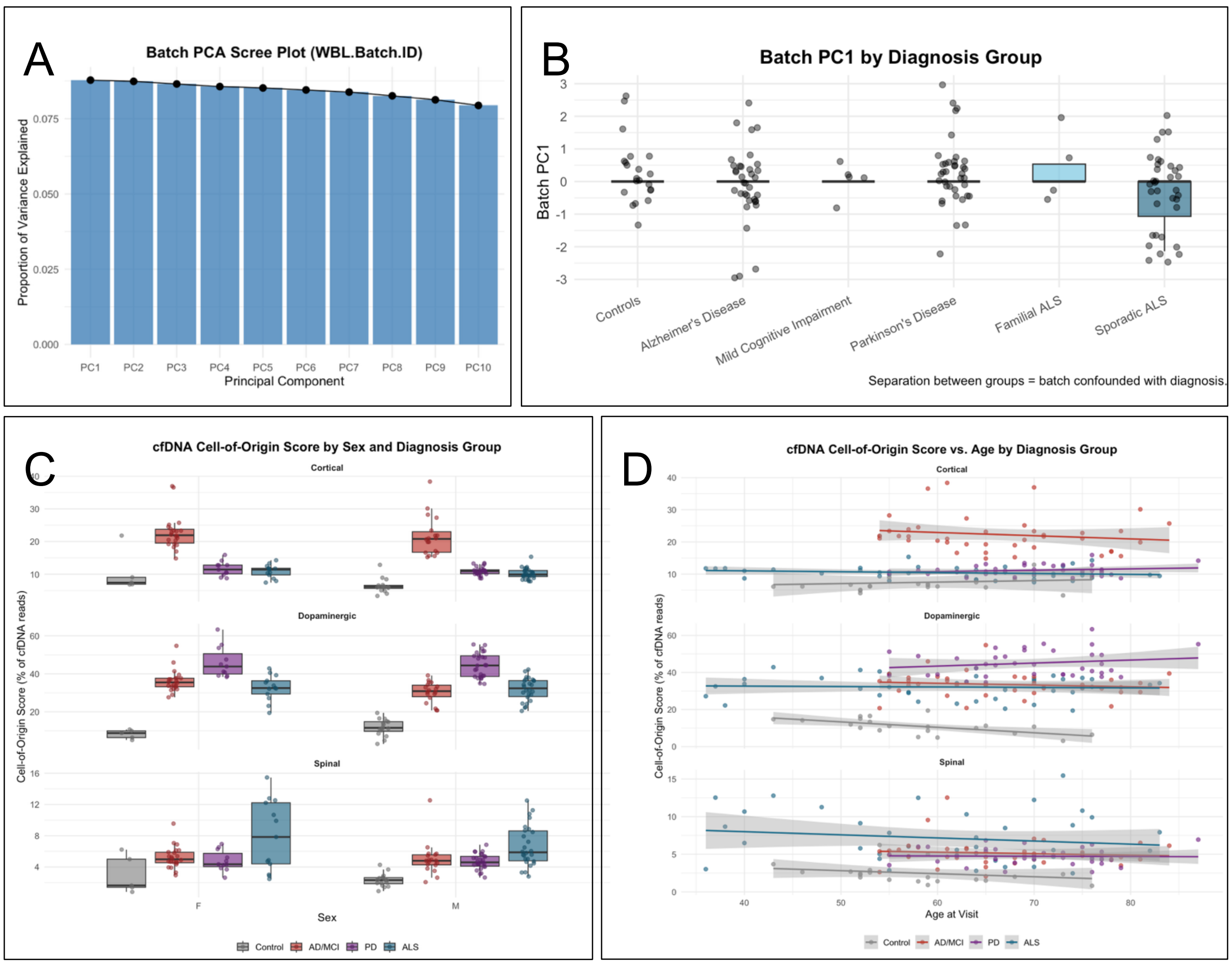

2.2 **Supplementary Figure 2. Read length distribution of dilution series datasets by cell type.** Histograms of read length distributions for cell type-derived reads before (A) and after (B) computational fragmentation. Post fragmentation profiles display read lengths that more closely simulate blood plasma cfDNA distributions.

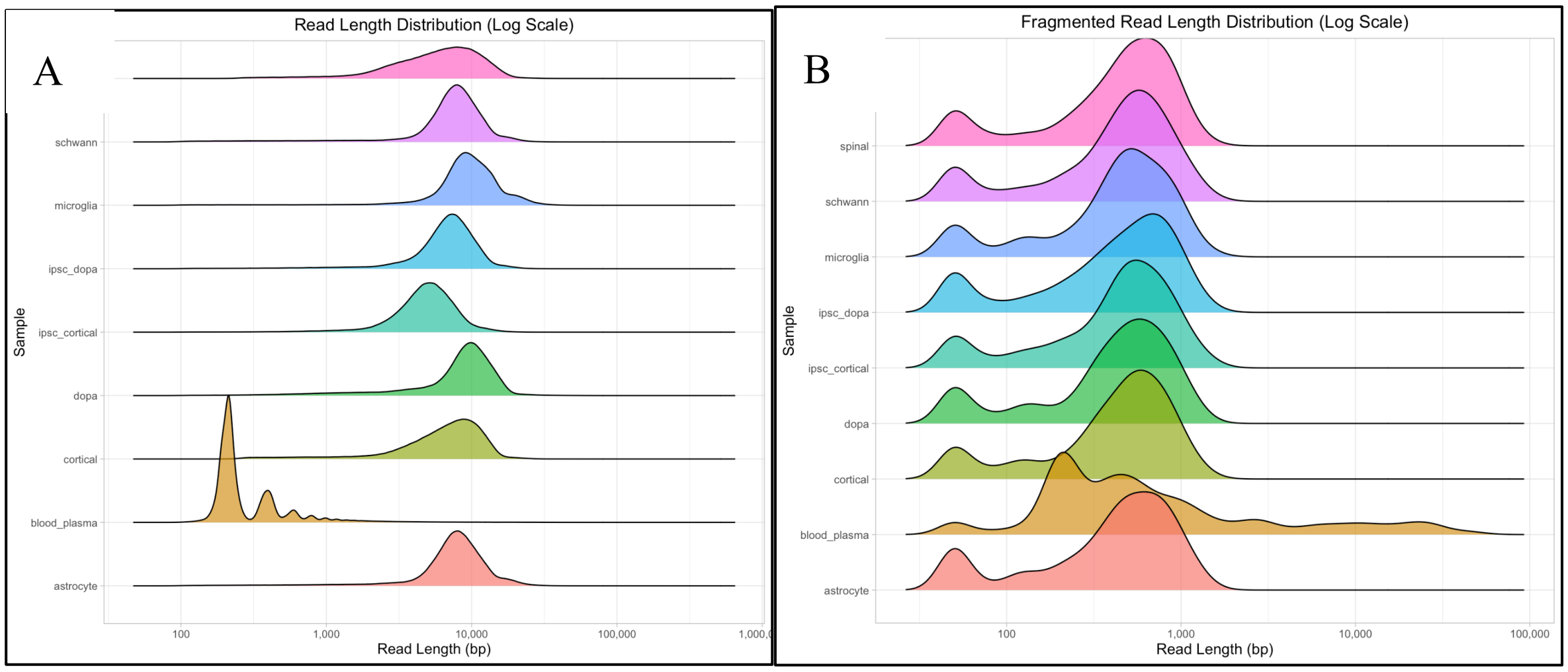

2.3 **Supplementary Figure 3. Primary cortical neuron DMRs specifically predict *in silico* cortical neuron DNA fractions.** Correlation plots show predicted versus true cortical neuron fractions for *in silico* mixtures of human primary cortical neuron-derived reads. The primary cortical neuron-trained classifier (A) demonstrated strongest correlation ($R^2 = 0.998$). In contrast, cell-of-origin classifiers trained on non-cortical neural cell-like differentially methylated regions (DMRs), including primary dopaminergic neurons (B), spinal motor neurons (C), astrocytes (D), Schwann cells (E), and microglia (F), and iPSC-derived neurons (G–I), showed no predictive power. Each point represents a single *in silico* dilution (~1,000,000 total reads), with nine fractional compositions (0–100% cell-type-derived reads) generated per cell type (Supplementary Table 3).

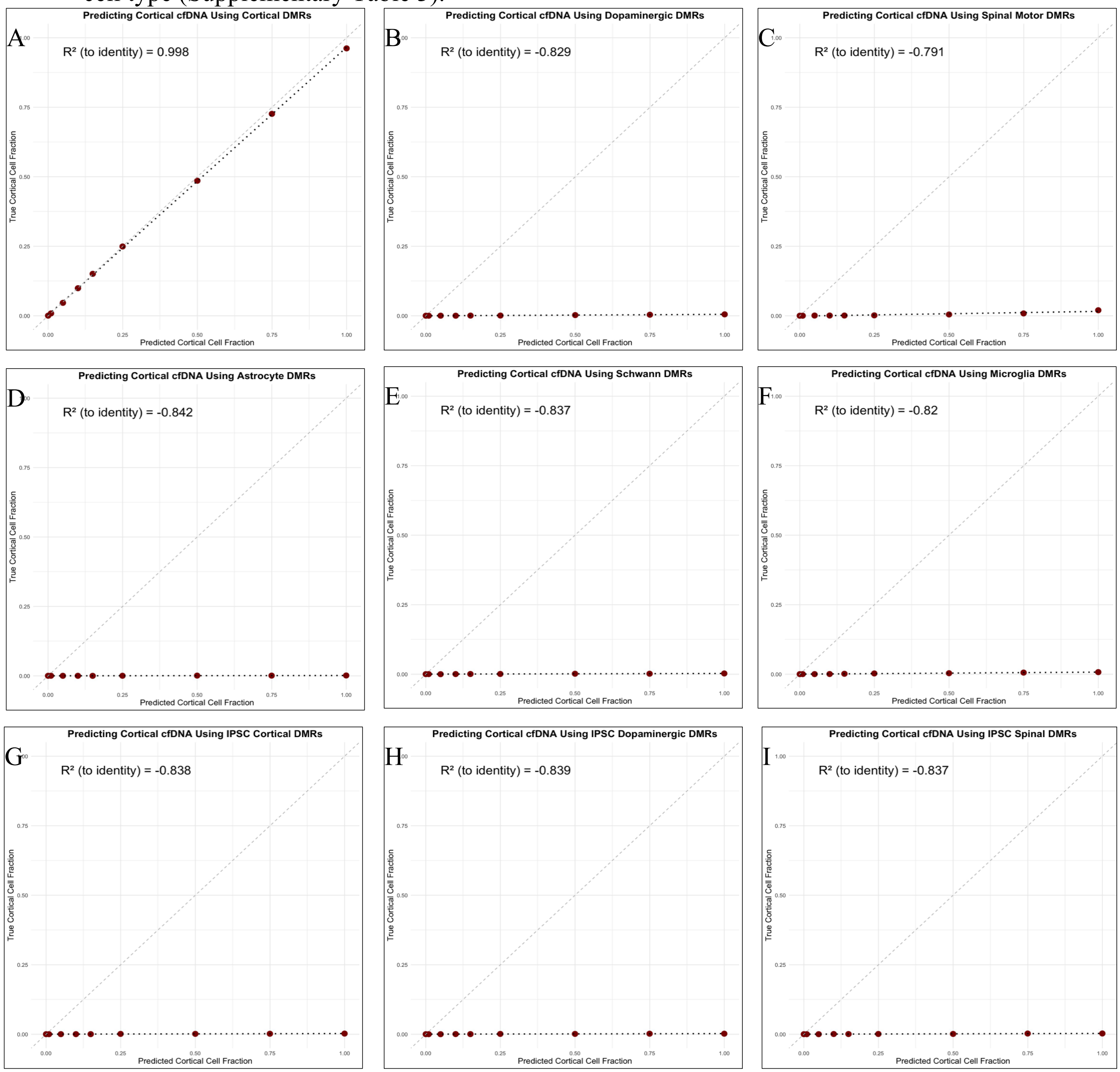

2.4 **Supplementary Figure 4. Primary dopaminergic neuron DMRs specifically predict in silico dopaminergic neuron DNA fractions.** Correlation plots show predicted versus true dopaminergic neuron fractions for *in silico* mixtures of human primary dopaminergic neuron-derived reads. The primary dopaminergic neuron-trained classifier (B) demonstrated strongest correlation ($R^2 = 0.993$). In contrast, cell-of-origin classifiers trained on non-dopaminergic neural cell-like differentially methylated regions (DMRs), including primary cortical neurons (A), spinal motor neurons (C), astrocytes (D), Schwann cells (E), and microglia (F), and iPSC-derived neurons (G–I), showed no predictive power. Each point represents a single *in silico* dilution (~1,000,000 total reads), with nine fractional compositions (0–100% cell-type-derived reads) generated per cell type (Supplementary Table 3).

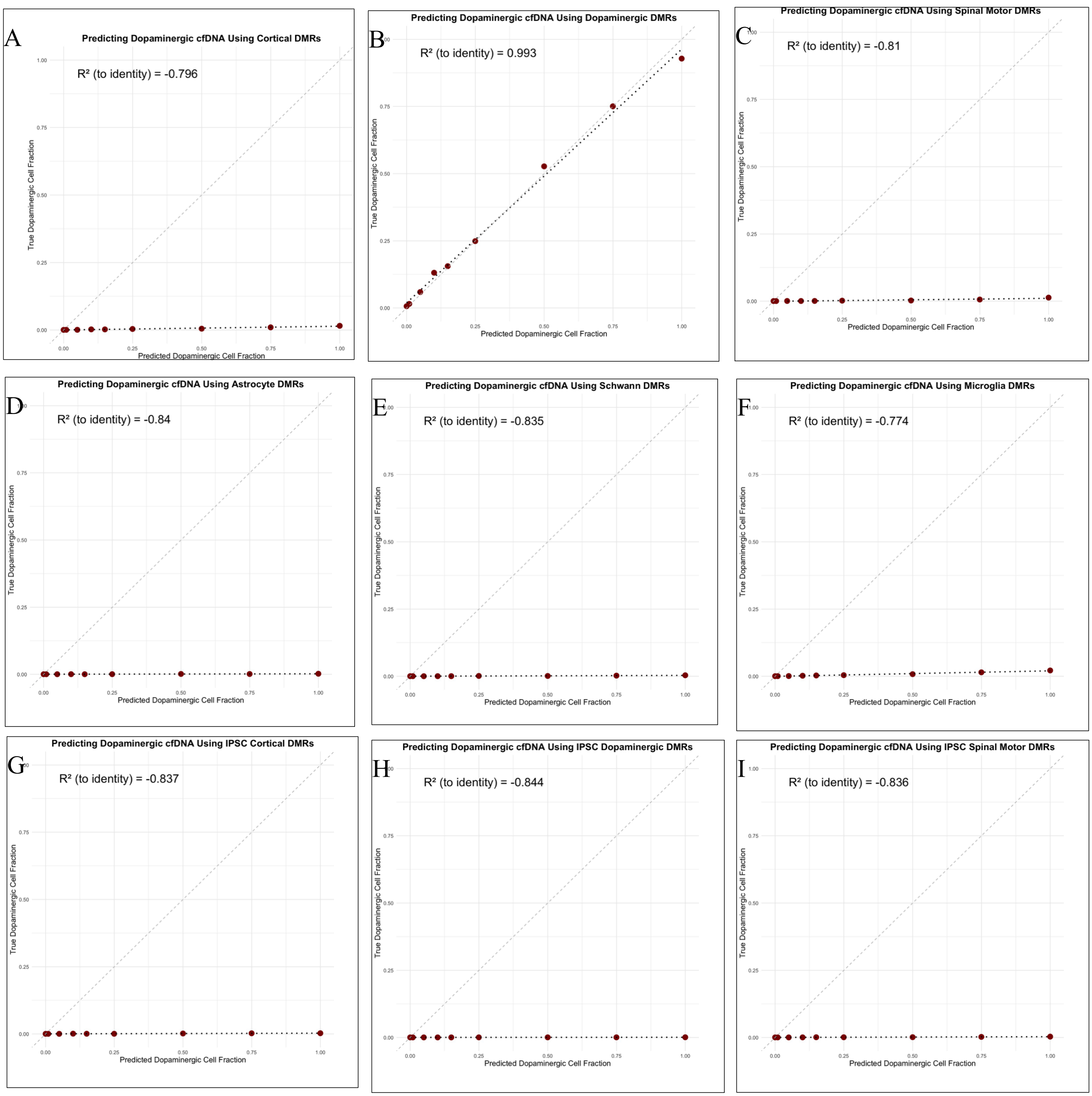

## 2.5 Supplementary Figure 5. Primary spinal motor neuron DMRs specifically predict *in silico* spinal motor neuron DNA fractions.

Correlation plots show predicted versus true spinal motor neuron fractions for *in silico* mixtures of human primary spinal motor neuron-derived reads. The spinal motor neuron-trained classifier (C) demonstrated highest correlation ($R^2 = 0.876$). In contrast, cell-of-origin classifiers trained on non-spinal motor neuron-like differentially methylated regions (DMRs), including primary cortical neurons (A), dopaminergic neurons (B), astrocytes (D), Schwann cells (E), and microglia (F), and iPSC-derived neurons (G–I), showed no predictive power. Each point represents a single in silico dilution (~1,000,000 total reads), with nine fractional compositions (0–100% cell-type-derived reads) generated per cell type (Supplementary Table 3).

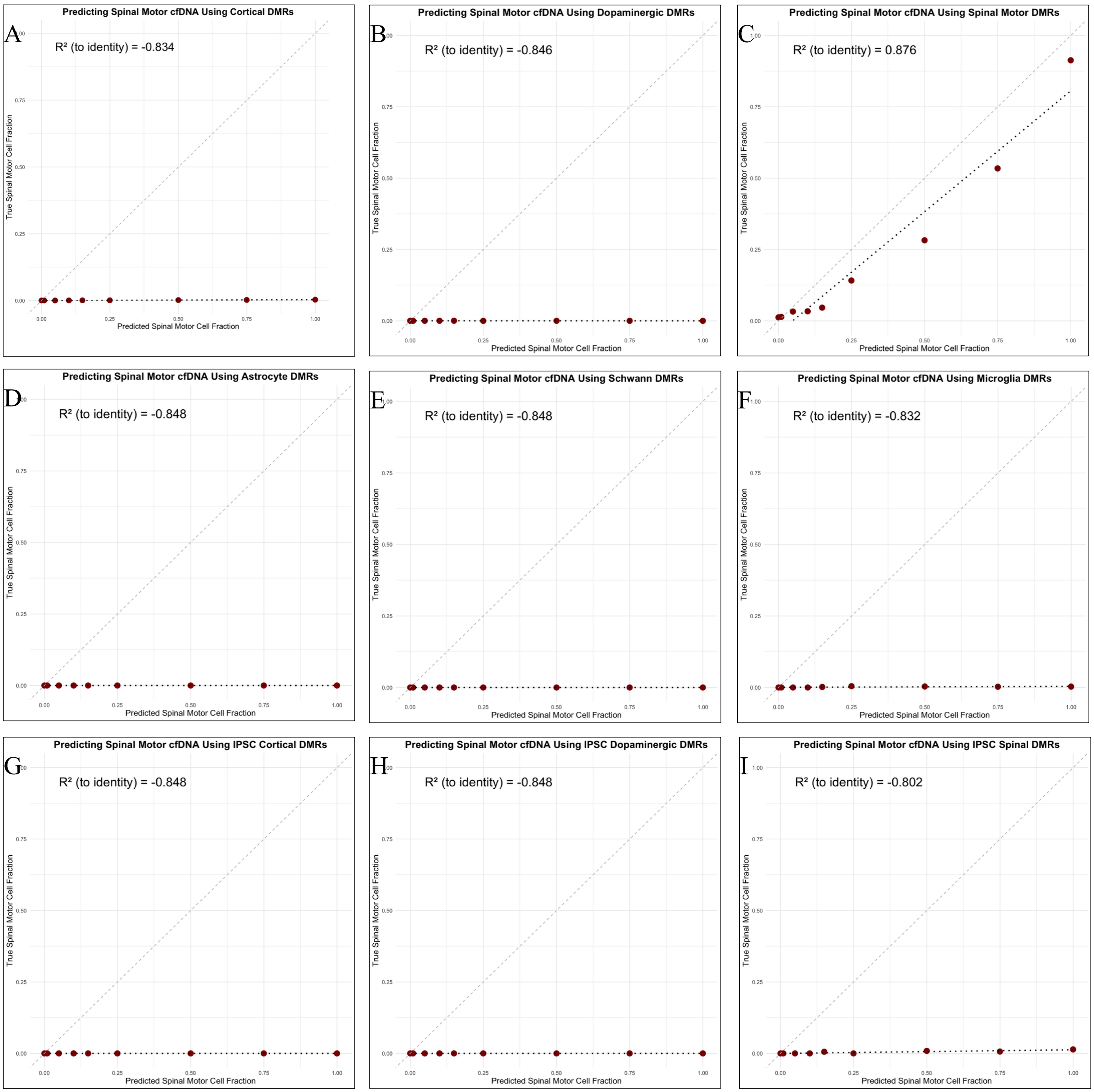

**2.6 Supplementary Figure 6. Primary astrocyte DMRs specifically predict *in silico* astrocyte DNA fractions.** Correlation plots show predicted versus true astrocyte fractions for *in silico* mixtures of human primary astrocyte-derived reads. The astrocyte-trained classifier (D) demonstrated strongest correlation ($R^2 = 0.993$). Cell-of-origin classifiers trained on non-astrocyte neural cell-like differentially methylated regions (DMRs), including primary cortical neurons (A), dopaminergic neurons (B), spinal motor neurons (C), Schwann cells (E), and microglia (F), and iPSC-derived neurons (G–I), showed no predictive power. Each point represents a single *in silico* dilution (~1,000,000 total reads), with nine fractional compositions (0–100% cell-type-derived reads) generated per cell type (Supplementary Table 3).

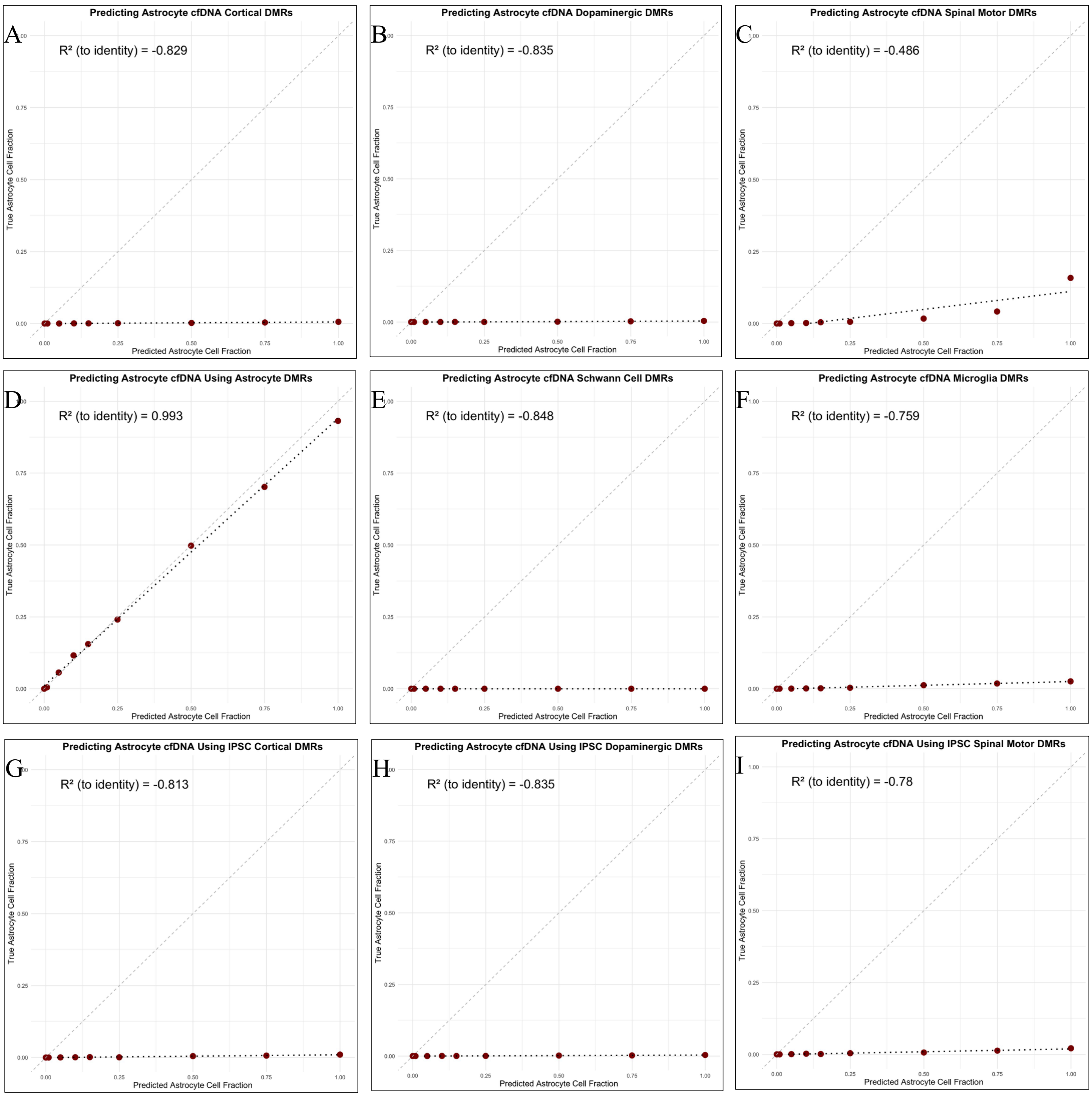

## 2.7 Supplementary Figure 7. Primary Schwann cell DMRs specifically predict *in silico* Schwann cell DNA fractions.

**Supplementary Figure 7. Primary Schwann cell DMRs specifically predict *in silico* Schwann cell DNA fractions.** Correlation plots show predicted versus true Schwann cell fractions for *in silico* mixtures of human primary Schwann cell-derived reads. The Schwann cell-trained classifier (E) demonstrated strongest correlation ($R^2 = 0.986$). In contrast, cell-of-origin classifiers trained on non-Schwann cell-like differentially methylated regions (DMRs), including primary cortical neurons (A), dopaminergic neurons (B), spinal motor neurons (C), astrocytes (D), and microglia (F), and iPSC-derived neurons (G–I), showed no predictive power. Each point represents a single *in silico* dilution (~1,000,000 total reads), with nine fractional compositions (0–100% cell-type-derived reads) generated per cell type (Supplementary Table 3).

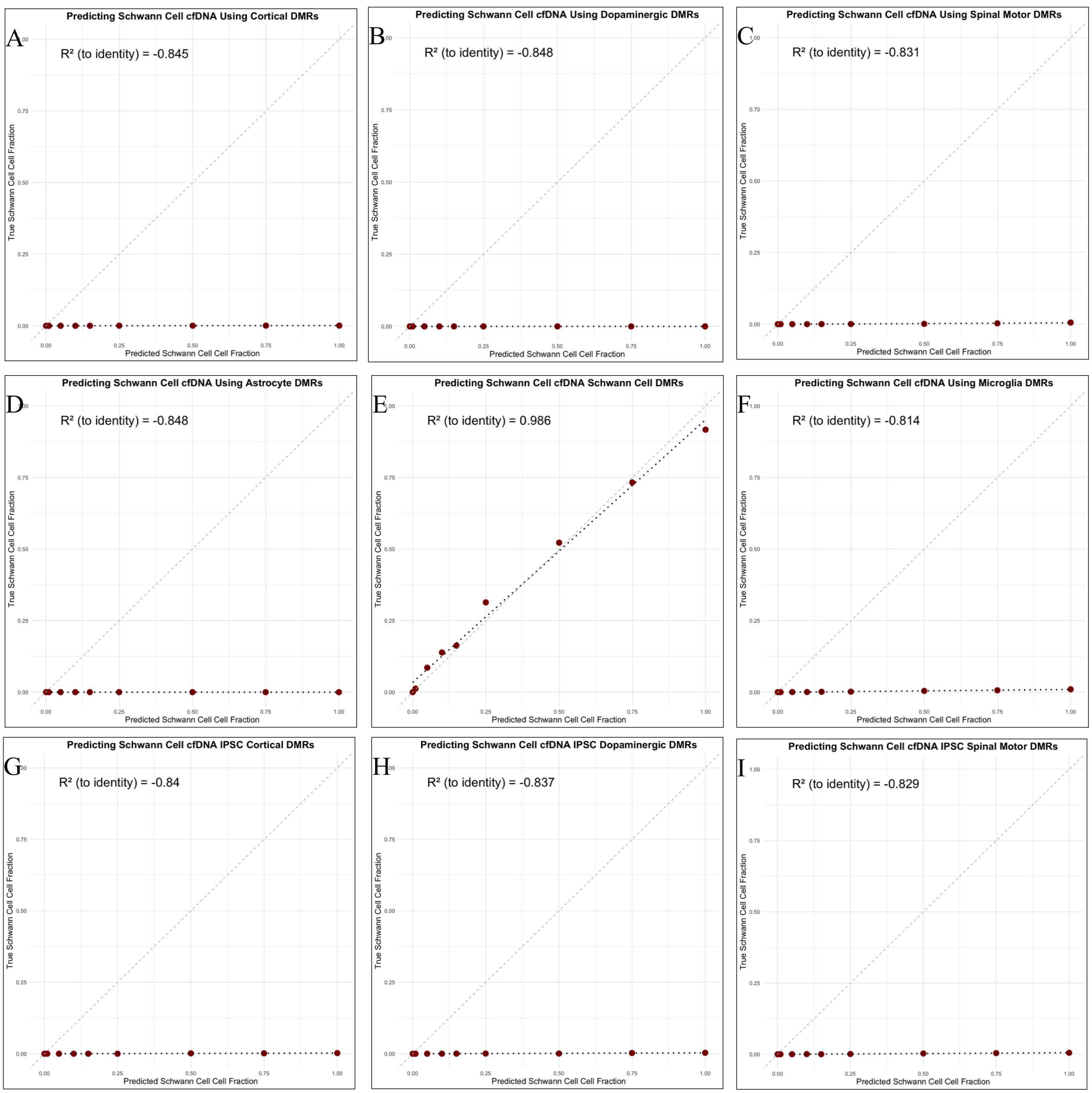

2.8 **Supplementary Figure 8. Primary microglia DMRs specifically predict *in silico* microglia DNA fractions.** Correlation plots show predicted versus true microglia fractions for *in silico* mixtures of human primary microglia-derived reads. The microglia-trained classifier (F) demonstrated strongest correlation ($R^2 = 0.992$). Cell-of-origin classifiers trained on non-microglia neural cell-like differentially methylated regions (DMRs), including primary cortical neurons (A), dopaminergic neurons (B), spinal motor neurons (C), astrocytes (D), and Schwann cells (E), and iPSC-derived neurons (G–I), showed no predictive power. Each point represents a single *in silico* dilution (~1,000,000 total reads), with nine fractional compositions (0–100% cell-type-derived reads) generated per cell type (Supplementary Table 3).

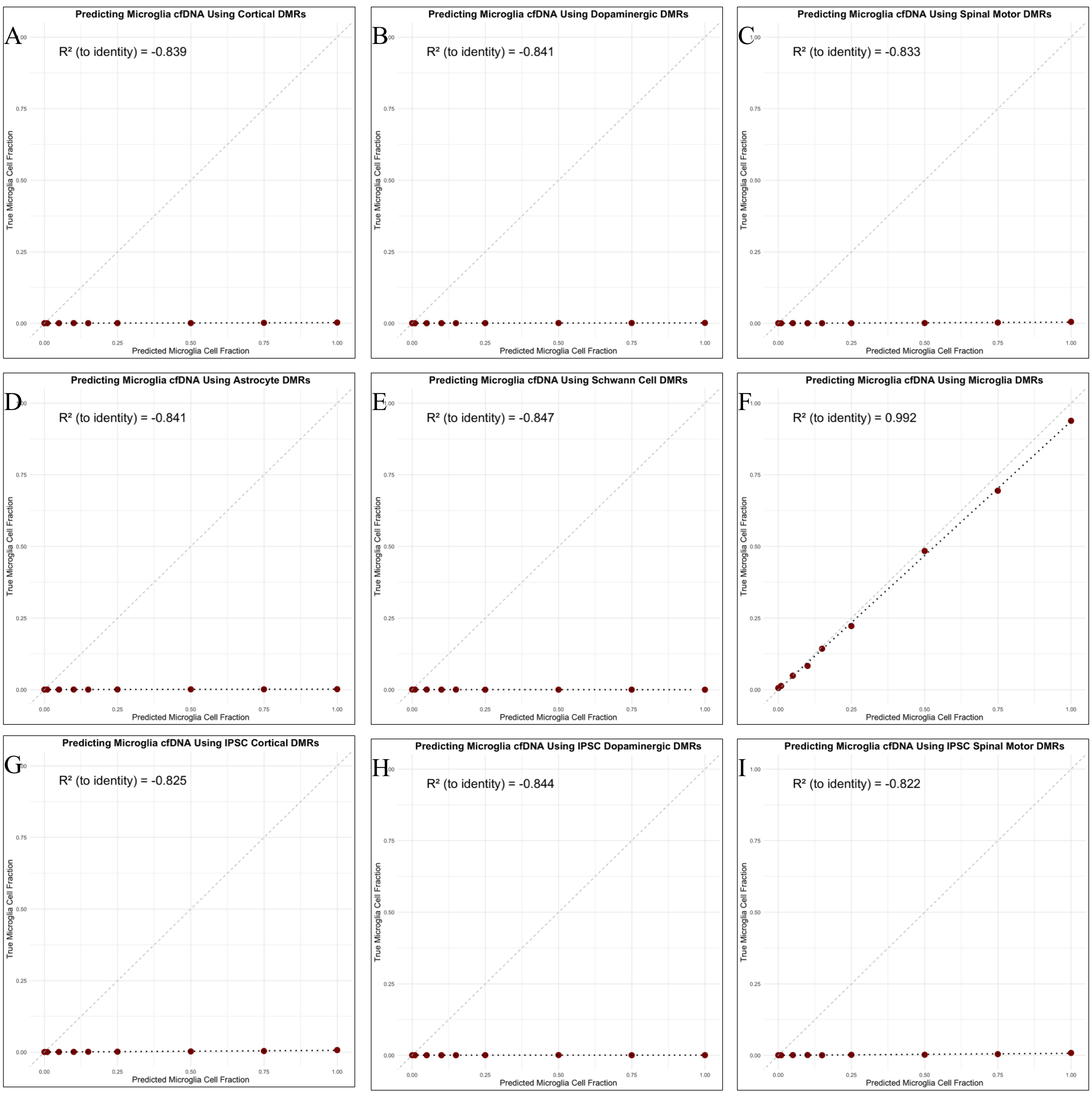

## 2.9 Supplementary Figure 9. Primary cortical neuron DMRs do not predict *in silico* iPSC cortical neuron DNA fractions.

**Supplementary Figure 9. Primary cortical neuron DMRs do not predict *in silico* iPSC cortical neuron DNA fractions.** Correlation plots show predicted versus true cortical neuron fractions for in silico mixtures of human iPSC cortical neuron reads. The iPSC cortical neuron-trained classifier accurately predicted iPSC cortical neuron fractions (G; R2 = 0.946) while the primary cortical neuron-trained classifier failed to predict iPSC cortical neuron fractions (A; R2 = -0.836). Cell-of-origin classifiers trained on primary neuronal (A–C) and glial (D–F) DMRs showed no predictive power when applied to the iPSC-derived reads, suggesting iPSC neuron-derived methylation signatures do not recapitulate primary neuronal methylation patterns required for cell-of-origin classification. Each point represents a single in silico dilution (~1,000,000 total reads), with nine fractional compositions (0–100% cell-type-derived reads) generated per cell type (Supplementary Table 3).

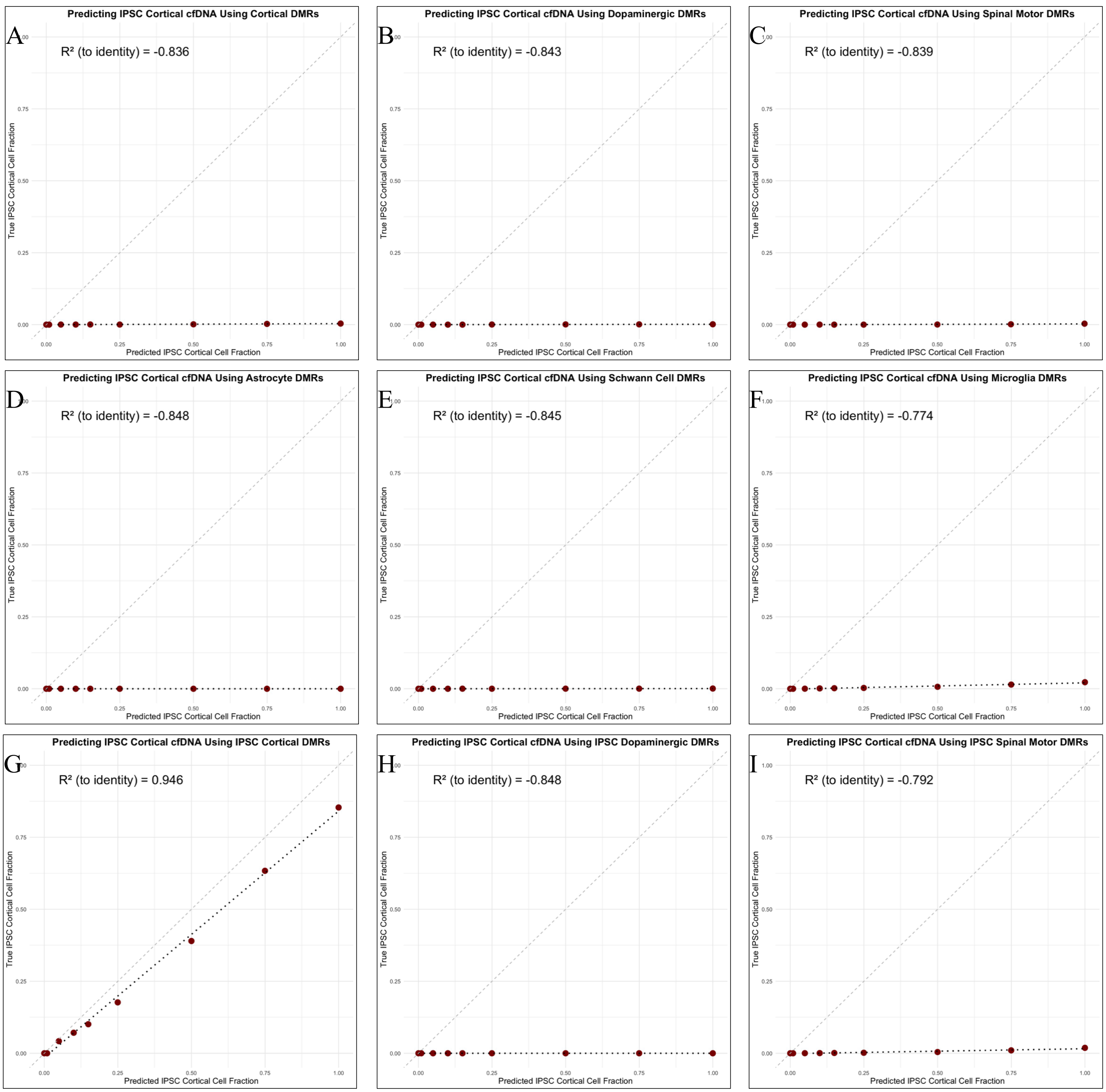

2.10 **Supplementary Figure 10. Primary dopaminergic neuron DMRs do not predict in silico iPSC dopaminergic neuron DNA fractions.** Correlation plots show predicted versus true dopaminergic neuron fractions for *in silico* mixtures of human iPSC dopaminergic neuron-derived reads. The iPSC dopaminergic neuron-trained classifier accurately predicted iPSC dopaminergic neuron fractions (H; $R^2 = 0.994$) while the primary dopaminergic neuron-trained classifier failed to predict iPSC dopaminergic neuron fractions (B; $R^2 = -0.832$). Cell-of-origin classifiers trained on primary neuronal (A–C) and glial (D–F) DMRs showed no predictive power when applied to the iPSC-derived reads, suggesting that iPSC dopaminergic neuron methylation signatures do not recapitulate primary dopaminergic neuronal methylation patterns required for cell-of-origin classification. Each point represents a single *in silico* dilution (~1,000,000 total reads), with nine fractional compositions (0–100% cell-type-derived reads) generated per cell type (Supplementary Table 3).

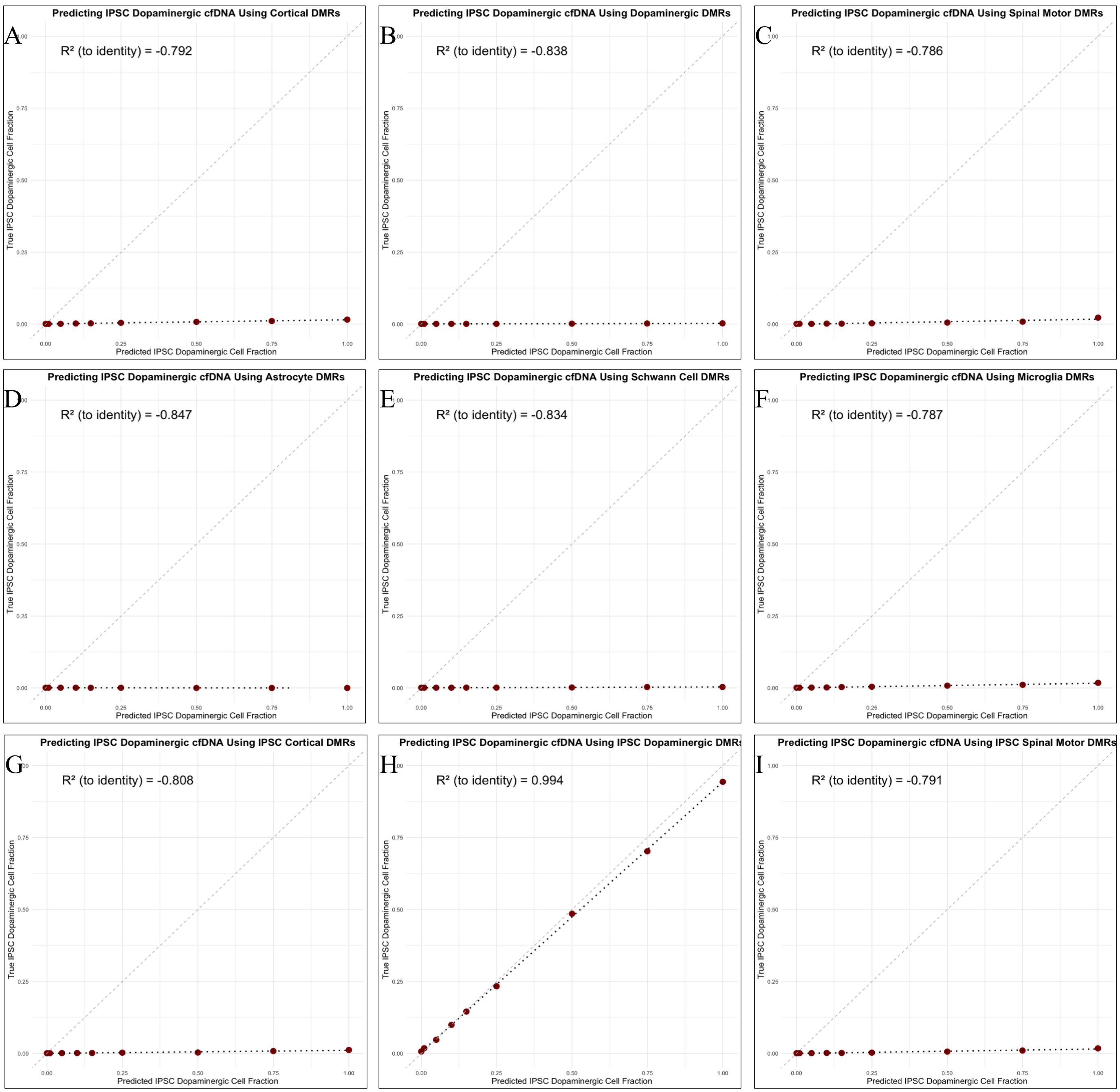

## 2.11 Supplementary Figure 11. Primary spinal motor neuron DMRs do not predict in silico iPSC spinal motor neuron DNA fractions.

**Supplementary Figure 11. Primary spinal motor neuron DMRs do not predict in silico iPSC spinal motor neuron DNA fractions.** Correlation plots show predicted versus true spinal motor neuron fractions for *in silico* mixtures of human iPSC spinal motor neuron-derived reads. The iPSC spinal motor neuron-trained classifier accurately predicted iPSC spinal motor neuron fractions (I; $R^2 = 0.982$), whereas the primary spinal motor neuron-trained classifier failed to predict iPSC fractions (C; $R^2 = -0.848$). Cell-of-origin classifiers trained on primary neuronal (A–C) and glial (D–F) DMRs showed no predictive power when applied to the iPSC-derived reads, suggesting that iPSC spinal motor neuron methylation signatures do not recapitulate primary spinal motor neuron methylation patterns required for cell-of-origin classification. Each point represents a single *in silico* dilution (~1,000,000 total reads), with nine fractional compositions (0–100% cell-type-derived reads) generated per cell type (Supplementary Table 3).

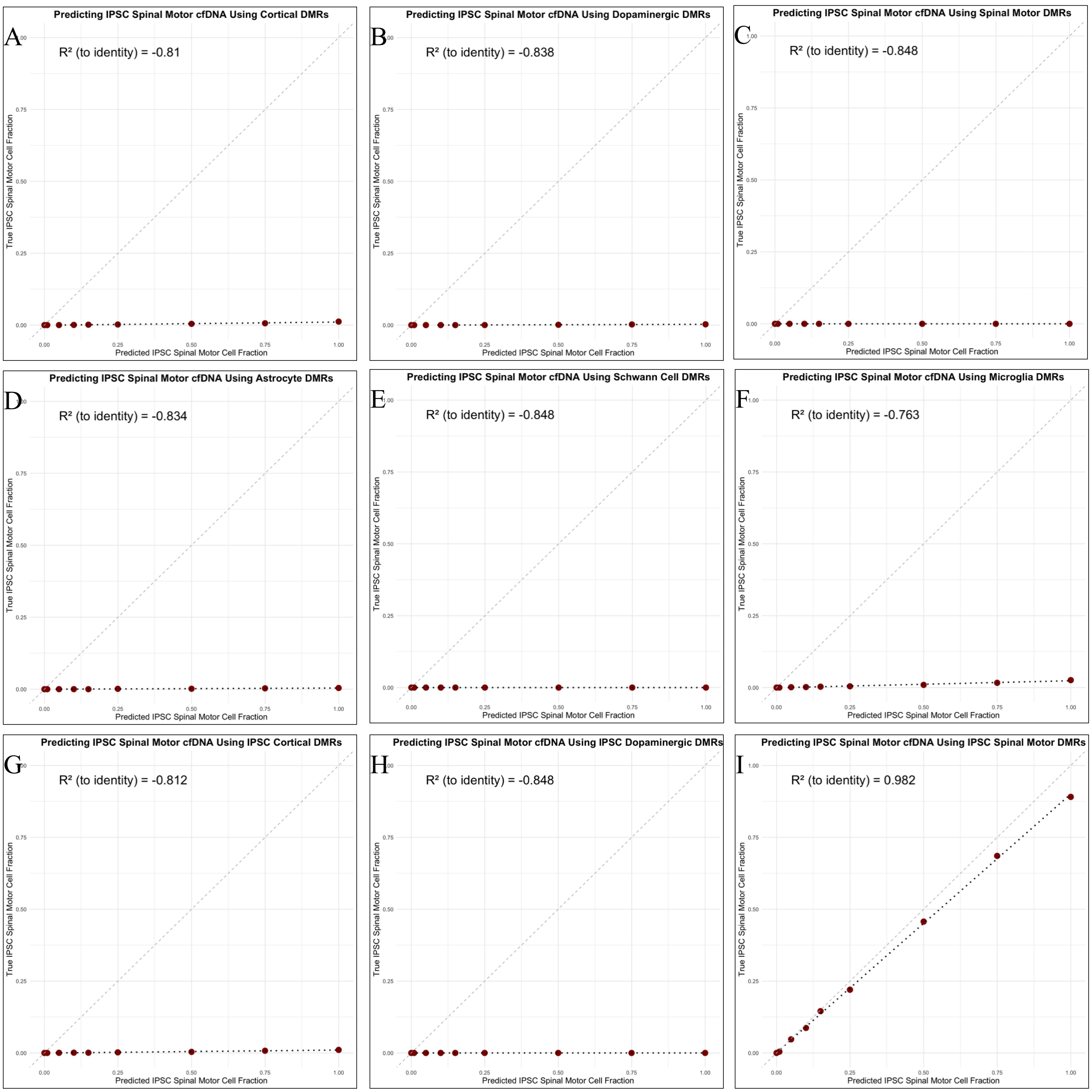

2.12 **Supplementary Figure 12. Overlayed ROC curves for covariates-only, methylation-only, and combined models in AD vs control, PD vs control, and ALS vs control.** ROC curves are shown for (A) Alzheimer's disease versus control, (B) Parkinson's disease versus control, and (C) ALS versus control. Covariates included age, sex, and flow cell.

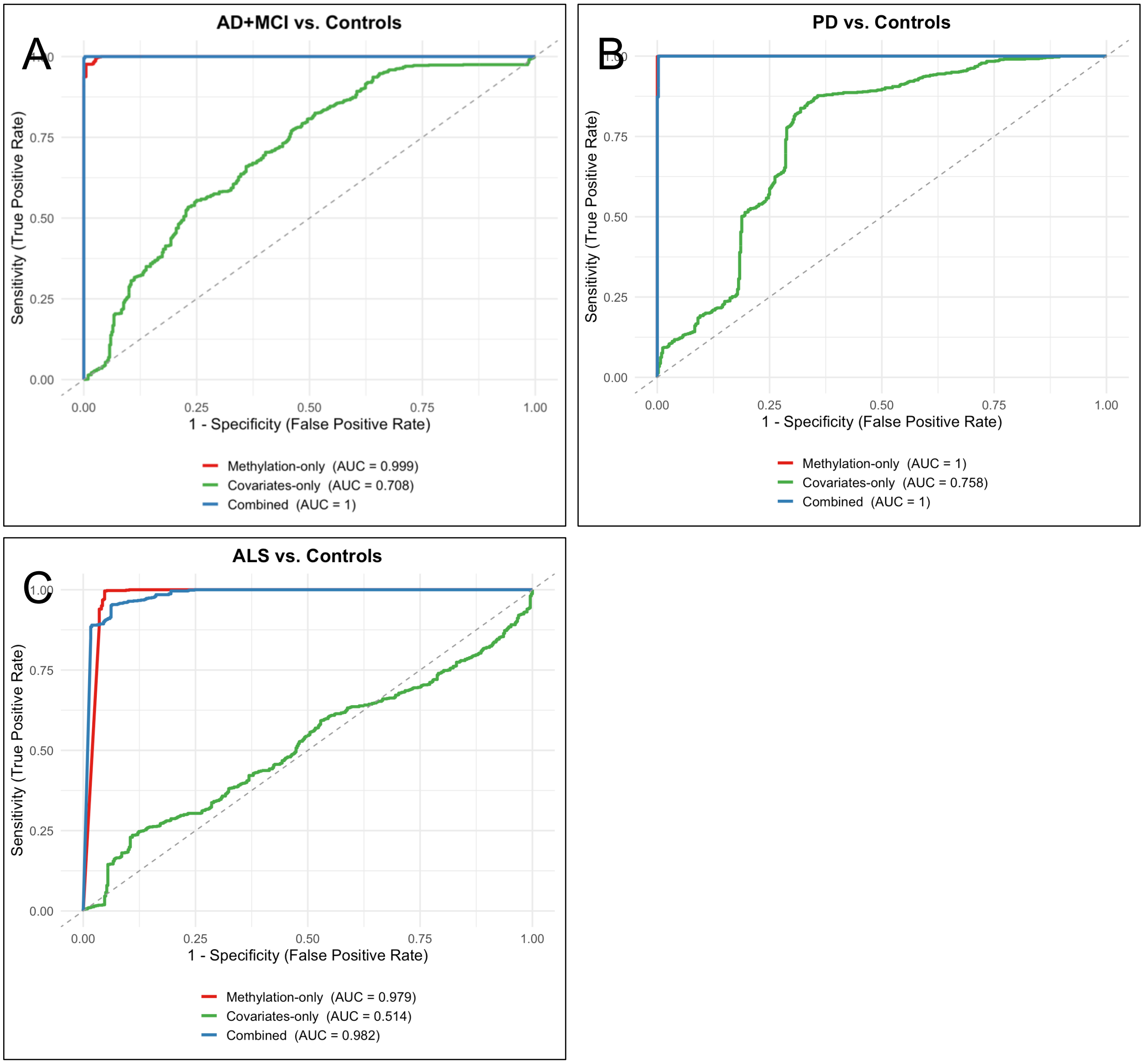

2.13 **Supplementary Figure 13. Orthogonal Alzheimer's disease biomarkers in the study cohort.** Box plots display raw protein biomarker concentrations and cognitive scores accompanying the blood plasma samples analyzed in this study. Shown are (A) amyloid-β 1–38, (B) amyloid-β 1–40, (C) amyloid-β 1–42, (D) the amyloid-β 1–42/1–40 ratio, (E) total tau, and (F) Mini-Mental State Examination (MMSE) scores across individuals with MCI (n = 3), Alzheimer's disease (n = 31), Parkinson's disease (n = 45), and ALS (n = 52. These measurements are presented as an ancillary clinical comparator and were not reprocessed through proprietary diagnostic pipelines, providing contextual comparison.

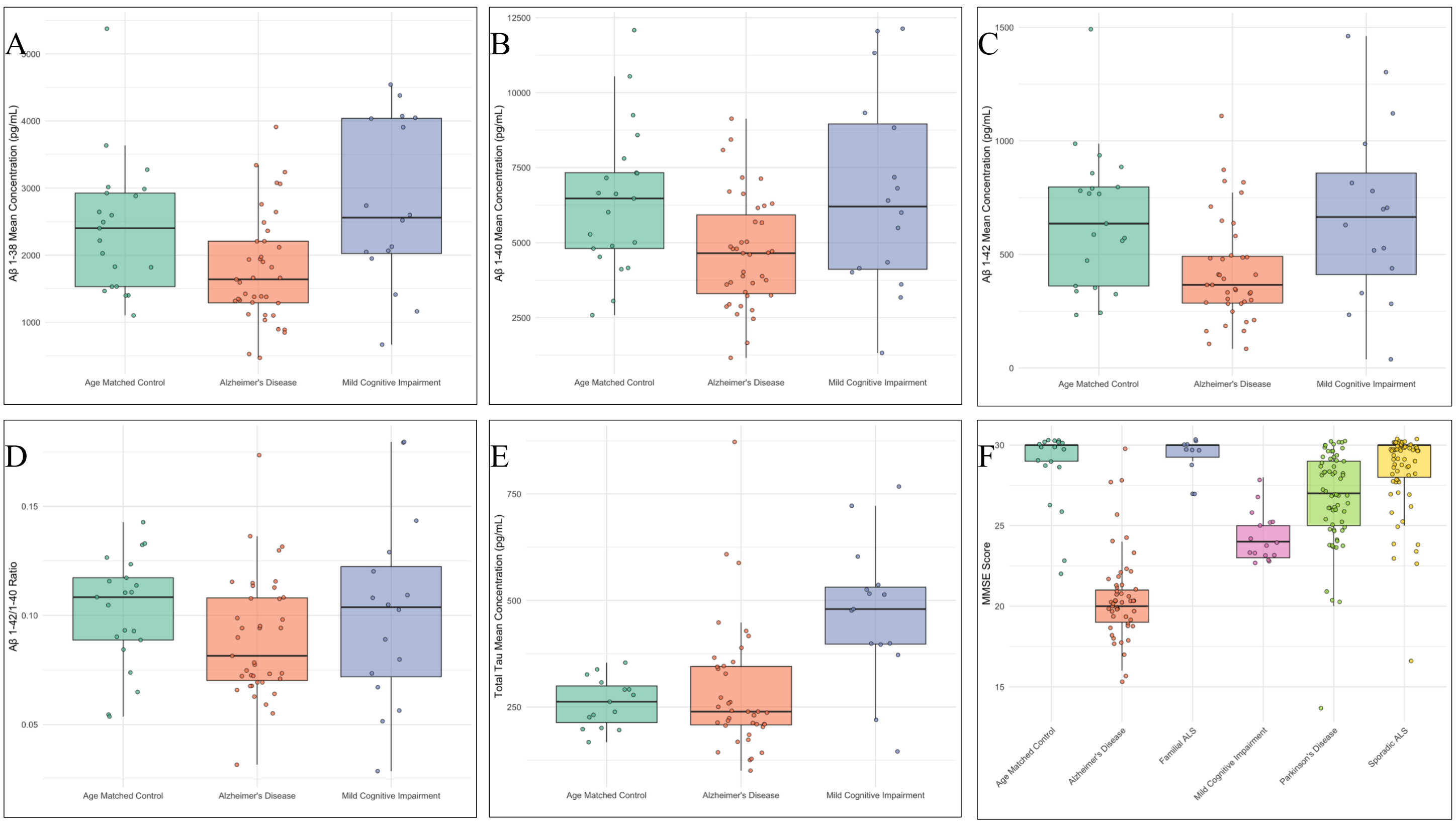

2.14 **Supplementary Figure 14. Non-disease-relevant cell classifiers do not show disease-associated cfDNA signals.** Box plots display the relative percentage of cfDNA reads classified to primary glial cell and iPSC glial cell types across all clinical blood plasma cohorts, including healthy controls (n = 21), Alzheimer's disease (n = 31), mild cognitive impairment (n = 3), Parkinson's disease (n = 45), familial ALS (n = 45), and sporadic ALS (n = 7). Shown are classifiers for primary astrocytes (A), Schwann cells (B), and microglia (C), and iPSC-derived cortical neurons (D), dopaminergic neurons (E), and spinal motor neurons (F).

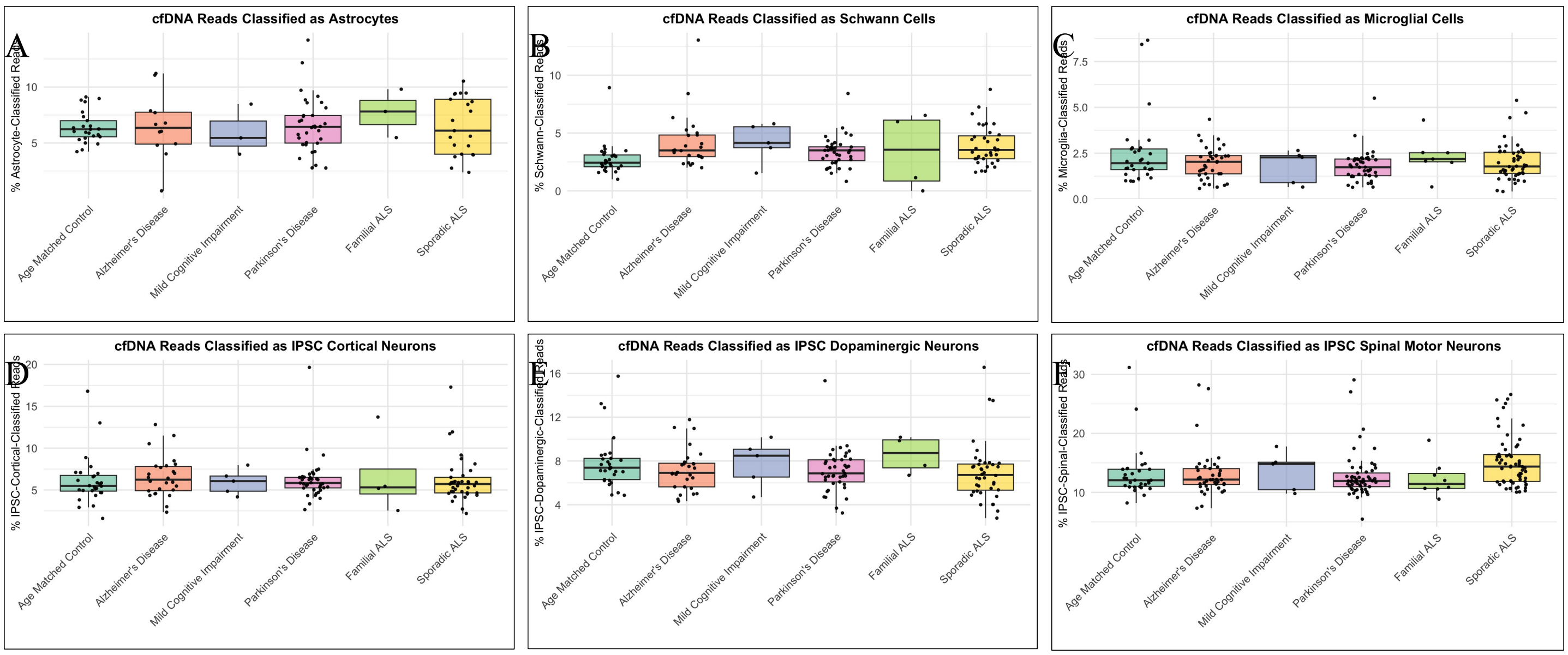

2.15 **Supplementary Figure 15. iPSC neuron DNA classifiers do not predict Alzheimer's disease.** Receiver operating characteristic (ROC) curves assessed the performance of iPSC-derived neuron cell-of-origin classifiers applied to blood plasma from healthy controls and patients with Alzheimer's disease (AD) or other neurodegenerative diseases (NDDs), including Parkinson's disease and amyotrophic lateral sclerosis. Classifiers for iPSC-derived cortical (A,E), dopaminergic (B,F), and spinal motor (C,G) neuron DNA were evaluated individually (A–C, E–D) and in combination (D, H). Panels compare classification of AD versus controls (A–D) and versus other NDDs (E–H). Across all analyses, iPSC-derived classifiers showed poor discriminative performance, underscoring their limited utility for predicting AD relative to primary neuron-based models.

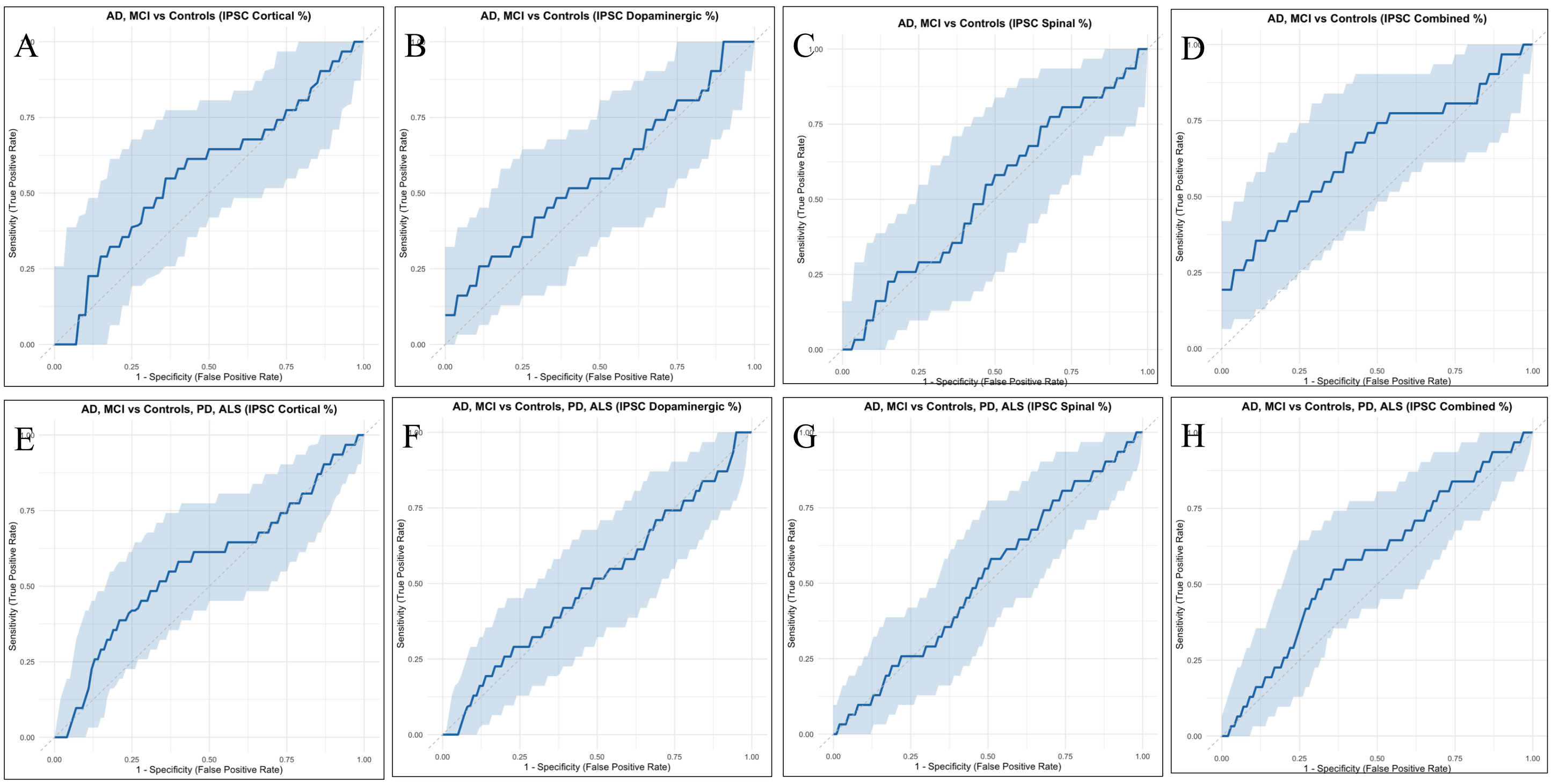


| AD vs Age Matched Control | Cortical % | Dopaminergic % | Spinal % | Combined Cell %'s |
|---|---|---|---|---|
| AUC | 0.5714 | 0.5622 | 0.5265 | 0.650 |
| AUC 95% CI Lower | 0.4217 | 0.4133 | 0.3756 | 0.5087 |
| AUC 95% CI Upper | 0.7212 | 0.7111 | 0.6774 | 0.7931 |
| Sensitivity | 0.5806 | 0.5161 | 0.1935 | 0.7742 |
| Specificity | 0.6429 | 0.6786 | 0.6786 | 0.5357 |
| Positive Predictive Value | 0.6429 | 0.64 | 0.4 | 0.6486 |
| Negative Predictive Value | 0.5806 | 0.5588 | 0.4318 | 0.6818 |
| Accuracy | 0.6102 | 0.5932 | 0.4237 | 0.661 |
| Balanced Accuracy | 0.6118 | 0.5974 | 0.4361 | 0.655 |
| Optimal Threshold | 0.5206 | 0.5432 | 0.5298 | 0.4898 |

| AD vs Controls + Other | Cortical % | Dopaminergic % | Spinal % | Combined Cell %'s |
|---|---|---|---|---|
| AUC | 0.5688 | 0.504 | 0.5192 | 0.58 |
| AUC 95% CI Lower | 0.445 | 0.3838 | 0.4036 | 0.4635 |
| AUC 95% CI Upper | 0.6926 | 0.6241 | 0.6347 | 0.6965 |
| Sensitivity | 0.5806 | 0.2903 | 0.5806 | 0.5161 |
| Specificity | 0.6132 | 0.8019 | 0.5189 | 0.6981 |
| Positive Predictive Value | 0.3051 | 0.3 | 0.2609 | 0.3333 |
| Negative Predictive Value | 0.8333 | 0.7944 | 0.8088 | 0.8315 |
| Accuracy | 0.6058 | 0.6861 | 0.5328 | 0.6569 |
| Balanced Accuracy | 0.5969 | 0.5461 | 0.5498 | 0.6071 |
| Optimal Threshold | 0.223 | 0.232 | 0.2237 | 0.2351 |

2.16 **Supplementary Figure 16. iPSC neuron DNA classifiers do not predict Parkinson's disease.** Receiver operating characteristic (ROC) curves assessed the performance of iPSC-derived neuron cell-of-origin classifiers applied to blood plasma from healthy controls and patients with Parkinson's disease (PD) or other neurodegenerative diseases (NDDs), including Alzheimer's disease and amyotrophic lateral sclerosis. Classifiers for iPSC-derived cortical (A,E), dopaminergic (B,F), and spinal motor (C,G) neuron DNA were evaluated individually (A–C, E–D) and in combination (D, H). Panels compare classification of PD versus controls (A–D) and versus other NDDs (E–H). Across all analyses, iPSC-derived classifiers showed poor discriminative performance, underscoring their limited utility for predicting PD relative to primary neuron-based models.

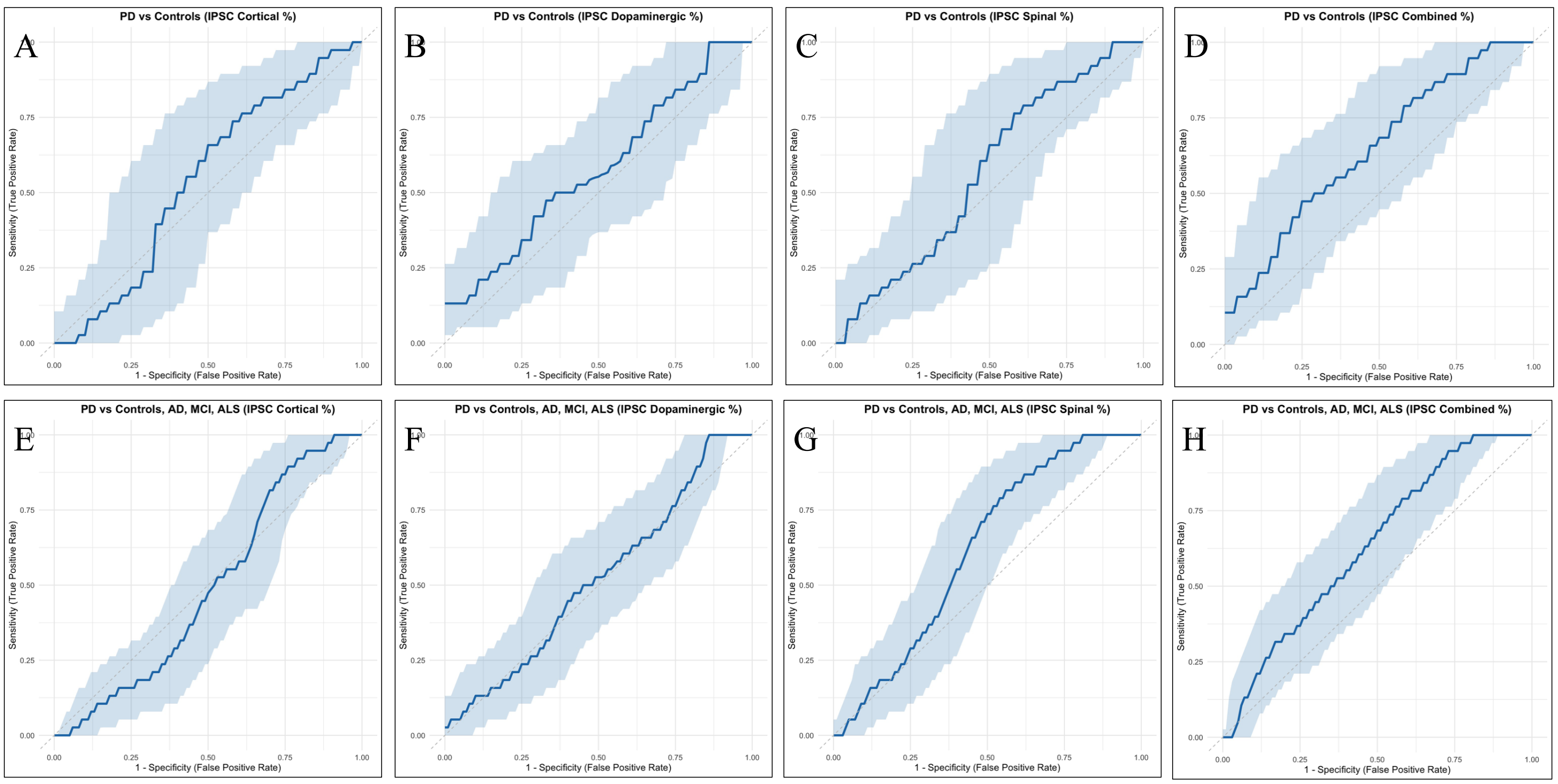


| PD vs Age Matched | Cortical % | Dopaminergic % | Spinal % | Combined Cell %'s |
|---|---|---|---|---|
| AUC | 0.5423 | 0.5733 | 0.5686 | 0.6438 |
| AUC 95% CI Lower | 0.3932 | 0.4318 | 0.4222 | 0.5077 |
| AUC 95% CI Upper | 0.6914 | 0.7148 | 0.715 | 0.7799 |
| Sensitivity | 0.2368 | 0.5 | 0.8158 | 0.4211 |
| Specificity | 0.5714 | 0.6786 | 0.3929 | 0.8214 |
| Positive Predictive | 0.4286 | 0.6786 | 0.6458 | 0.7619 |
| Negative Predictive | 0.3556 | 0.5 | 0.6111 | 0.5111 |
| Accuracy | 0.3788 | 0.5758 | 0.6364 | 0.5909 |
| Balanced Accuracy | 0.4041 | 0.5893 | 0.6043 | 0.6212 |

| | | | | |
|---|---|---|---|---|
| Optimal Threshold | 0.5853 | 0.6 | 0.5545 | 0.6574 |

| PD vs Controls + Other | Cortical % | Dopaminergic % | Spinal % | Combined Cell %'s |
|---|---|---|---|---|
| AUC | 0.4928 | 0.5223 | 0.6172 | 0.635 |
| AUC 95% CI Lower | 0.392 | 0.416 | 0.5215 | 0.537 |
| AUC 95% CI Upper | 0.5936 | 0.6287 | 0.713 | 0.7331 |
| Sensitivity | 0.9474 | 1 | 0.8158 | 0.9474 |
| Specificity | 0.202 | 0.1414 | 0.4545 | 0.2828 |
| Positive Predictive | 0.313 | 0.3089 | 0.3647 | 0.3364 |
| Negative Predictive | 0.9091 | 1 | 0.8654 | 0.9333 |
| Accuracy | 0.4088 | 0.3796 | 0.5547 | 0.4672 |
| Balanced Accuracy | 0.5747 | 0.5707 | 0.6352 | 0.6151 |
| Optimal Threshold | 0.2554 | 0.2238 | 0.2696 | 0.1891 |

## 2.17 **Supplementary Figure 17. iPSC neuron DNA classifiers do not predict amyotrophic lateral sclerosis. Receiver operating characteristic (ROC) curves assessed the performance of iPSC-**derived neuron cell-of-origin classifiers applied to blood plasma from healthy controls and patients with amyotrophic lateral sclerosis (ALS) or other neurodegenerative diseases (NDDs), including Alzheimer's disease and Parkinson's disease. Classifiers for iPSC-derived cortical (A,E), dopaminergic (B,F), and spinal motor (C,G) neuron DNA were evaluated individually (A–C, E–G) and in combination (D, H). Panels compare classification of ALS versus controls (A–D) and versus other NDDs (E–H). Across all analyses, iPSC-derived classifiers showed poor discriminative performance, underscoring their limited utility for predicting ALS relative to primary neuron–based models.

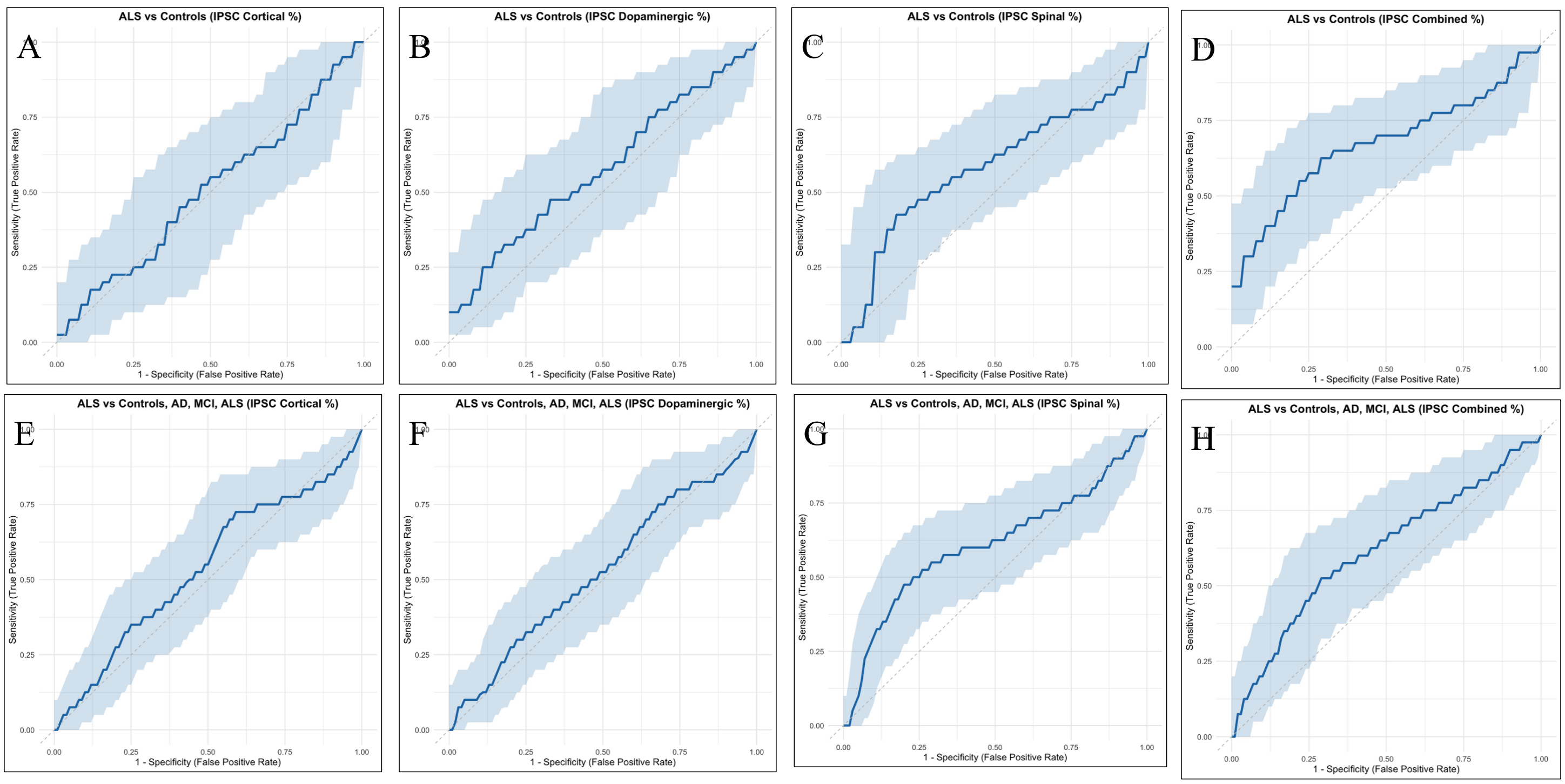


| ALS vs Age Matched | Cortical % | Dopaminergic % | Spinal % | Combined Cell %'s |
|---|---|---|---|---|
| AUC | 0.5027 | 0.5723 | 0.5812 | 0.666 |
| AUC 95% CI Lower | 0.3612 | 0.434 | 0.4424 | 0.5366 |
| AUC 95% CI Upper | 0.6441 | 0.7107 | 0.7201 | 0.7956 |
| Sensitivity | 0.45 | 0.3 | 0.475 | 0.675 |
| Specificity | 0.6429 | 0.8929 | 0.8214 | 0.6786 |
| Positive Predictive | 0.6429 | 0.8 | 0.7917 | 0.75 |
| Negative Predictive | 0.45 | 0.4717 | 0.5227 | 0.5938 |
| Accuracy | 0.5294 | 0.5441 | 0.6176 | 0.6765 |
| Balanced Accuracy | 0.5464 | 0.5964 | 0.6482 | 0.6768 |
| Optimal Threshold | 0.5869 | 0.6228 | 0.5914 | 0.5847 |

| ALS vs Controls + | Cortical % | Dopaminergic % | Spinal % | Combined Cell %'s |
|---|---|---|---|---|
| AUC | 0.5369 | 0.5299 | 0.608 | 0.6161 |
| AUC 95% CI Lower | 0.426 | 0.4196 | 0.4923 | 0.4998 |
| AUC 95% CI Upper | 0.6477 | 0.6402 | 0.7237 | 0.7324 |
| Sensitivity | 0.275 | 0.2 | 0.475 | 0.55 |
| Specificity | 0.5464 | 0.701 | 0.8144 | 0.7288 |
| Positive Predictive | 0.2 | 0.2162 | 0.5135 | 0.5789 |
| Negative Predictive | 0.6463 | 0.68 | 0.79 | 0.7049 |
| Accuracy | 0.4672 | 0.5547 | 0.7153 | 0.6566 |
| Balanced Accuracy | 0.4107 | 0.4505 | 0.6447 | 0.6394 |
| Optimal Threshold | 0.2912 | 0.2926 | 0.3055 | 0.4288 |

2.18 **Supplementary Figure 19. iPSC neuron DNA classifiers do not predict amyotrophic lateral sclerosis. Receiver operating characteristic (ROC) curves assessed the performance of iPSC-**derived neuron cell-of-origin classifiers applied to blood plasma from healthy controls and patients with amyotrophic lateral sclerosis (ALS) or other neurodegenerative diseases (NDDs), including Alzheimer's disease and Parkinson's disease. Classifiers for iPSC-derived cortical (A,E), dopaminergic (B,F), and spinal motor (C,G) neuron DNA were evaluated individually (A–C, E–G) and in combination (D, H). Panels compare classification of ALS versus controls (A–D) and versus other NDDs (E–H). Across all analyses, iPSC-derived classifiers showed poor discriminative performance, underscoring their limited utility for predicting ALS relative to primary neuron–based models.

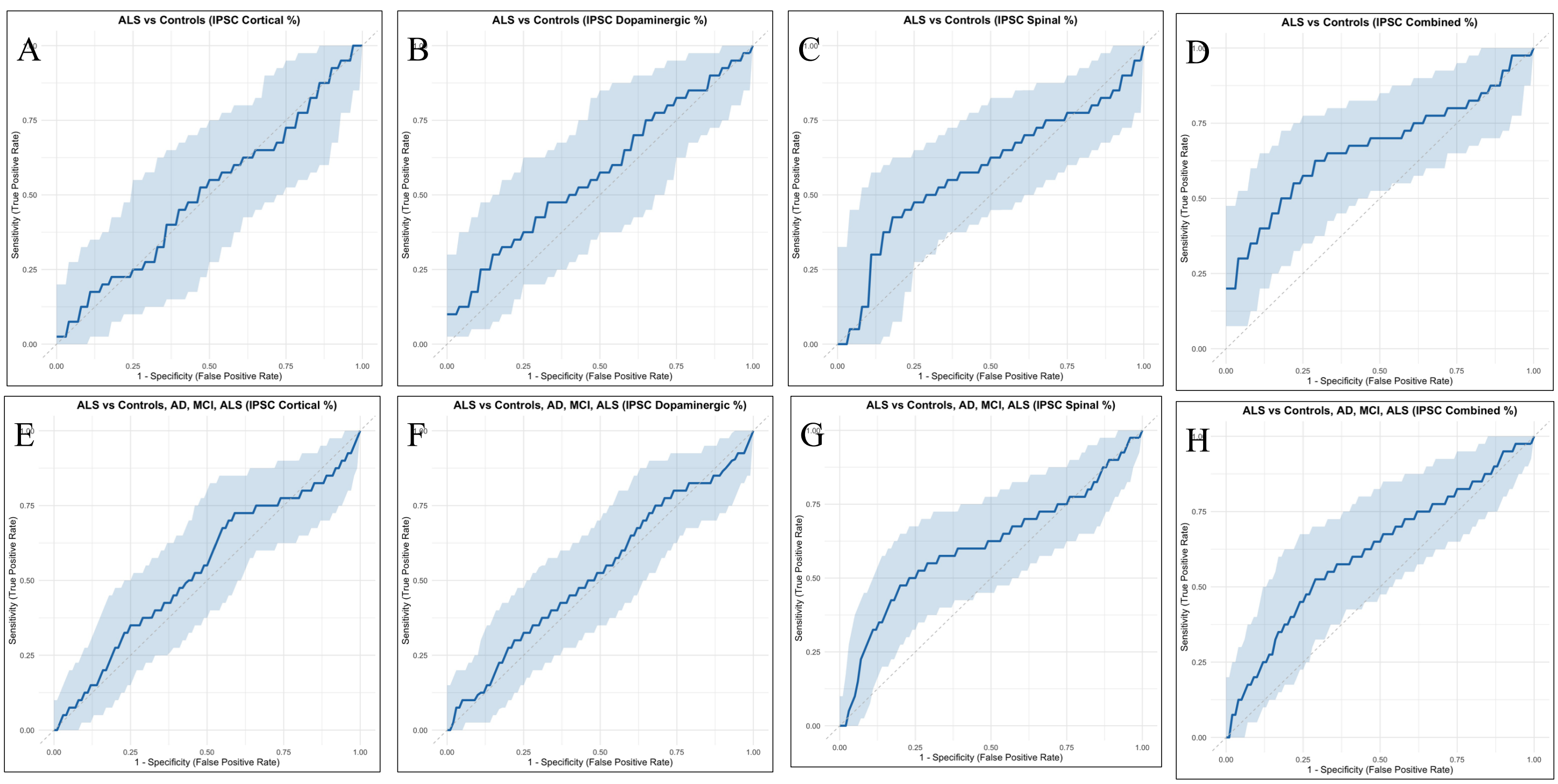


| ALS vs Age Matched | Cortical % | Dopaminergic % | Spinal % | Combined Cell %'s |
|---|---|---|---|---|
| AUC | 0.5027 | 0.5723 | 0.5812 | 0.666 |
| AUC 95% CI Lower | 0.3612 | 0.434 | 0.4424 | 0.5366 |
| AUC 95% CI Upper | 0.6441 | 0.7107 | 0.7201 | 0.7956 |
| Sensitivity | 0.45 | 0.3 | 0.475 | 0.675 |
| Specificity | 0.6429 | 0.8929 | 0.8214 | 0.6786 |
| Positive Predictive | 0.6429 | 0.8 | 0.7917 | 0.75 |
| Negative Predictive | 0.45 | 0.4717 | 0.5227 | 0.5938 |
| Accuracy | 0.5294 | 0.5441 | 0.6176 | 0.6765 |
| Balanced Accuracy | 0.5464 | 0.5964 | 0.6482 | 0.6768 |

| | | | | |
|---|---|---|---|---|
| Optimal Threshold | 0.5869 | 0.6228 | 0.5914 | 0.5847 |

| ALS vs Controls + | Cortical % | Dopaminergic % | Spinal % | Combined Cell %'s |
|---|---|---|---|---|
| AUC | 0.5369 | 0.5299 | 0.608 | 0.6161 |
| AUC 95% CI Lower | 0.426 | 0.4196 | 0.4923 | 0.4998 |
| AUC 95% CI Upper | 0.6477 | 0.6402 | 0.7237 | 0.7324 |
| Sensitivity | 0.275 | 0.2 | 0.475 | 0.55 |
| Specificity | 0.5464 | 0.701 | 0.8144 | 0.7288 |
| Positive Predictive | 0.2 | 0.2162 | 0.5135 | 0.5789 |
| Negative Predictive | 0.6463 | 0.68 | 0.79 | 0.7049 |
| Accuracy | 0.4672 | 0.5547 | 0.7153 | 0.6566 |
| Balanced Accuracy | 0.4107 | 0.4505 | 0.6447 | 0.6394 |
| Optimal Threshold | 0.2912 | 0.2926 | 0.3055 | 0.4288 |
